# Targeting bacterial influx pathways to defeat antibiotic resistance: fluorinated glucose-6-phosphate analogues revive fosfomycin efficacy

**DOI:** 10.1038/s44259-026-00239-y

**Published:** 2026-06-21

**Authors:** Kirsty Alexandra Teahan, Akhilesh Kumar Chaurasia, Joohyun Lee, Dong Hyeok Yoon, Jacob Bilsborrow, Tracey A. Newman, Nicholas D. Evans, Tae Hyung Won, Seung Seo Lee, Kyeong Kyu Kim

**Affiliations:** 1 School of Chemistry and Chemical Engineering, Faculty of Engineering and Physical Sciences, University of Southampton, Southampton, UK; 2 Department of Precision Medicine, Sungkyunkwan University School of Medicine, Suwon, South Korea; 3 Department of Pharmacology, Sungkyunkwan University School of Medicine, Suwon, South Korea; 4 School of Clinical and Experimental Sciences, Faculty of Medicine, University of Southampton, Southampton, UK; 5 Institute for Life Sciences, University of Southampton, Southampton, UK; 6 School of Human Development and Health, Faculty of Medicine, University of Southampton, Southampton, UK

**Keywords:** Biochemistry, Drug discovery, Microbiology

## Abstract

Current adjuvant strategies to combat antimicrobial resistance such as β-lactamase inhibitors and efflux pump blockers prevent antibiotic degradation or export. We introduce a fundamentally different approach: exploiting bacterial nutrient sensing to drive antibiotic influx and overwhelm resistance. Fosfomycin is transported into bacterial cytoplasm via the glucose-6-phosphate (G6P) channel UhpT, which is activated by G6P. We demonstrate that fluorinated glucose-6-phosphate analogues showed superior activation of UhpT, leading to higher fosfomycin influx. In *Escherichia coli*, 3-fluoro-D-glucopyranosyl-6-phosphate showed 128-fold potentiation of fosfomycin, reversed *fosA*-mediated fosfomycin resistance, and displayed profound suppression of resistance evolution. In *Staphylococcus aureus*, 4-fluoro-D-glucopyranosyl-6-phosphate achieved 32-fold potentiation and broke *fosB*-mediated resistance. Furthermore, co-administration of these analogues improved *Galleria mellonella* survival (75-80%) versus fosfomycin monotherapy (30–40%). We demonstrate this high potentiation effect is combination of metabolic stability, persistent extracellular presence and favorable binding to the sensor protein. This influx-targeting strategy establishes a new paradigm to revive legacy antibiotics against multidrug-resistant pathogens.

## Introduction

The discovery and deployment of antibiotics revolutionized modern medicine, drastically reducing mortality from bacterial infections. Yet, the golden age of antibiotics is fading, as antimicrobial resistance (AMR) escalates into a critical global health threat. In the United States alone, over 2 million hospital-acquired infections occur annually, incurring approximately $20 billion in excess healthcare costs^1^ and globally, in 2021 alone it was estimated that 4.7 million deaths were associated with bacterial AMR, and among them, 1.14 million deaths attributable to bacterial AMR^2^. Alarmingly, a UK government report projected AMR-related deaths could reach 10 million per year by 2050, surpassing the global cancer burden. More recently, a comprehensive 2019 analysis estimated nearly 5 million deaths associated with bacterial AMR worldwide, including 1.27 million directly attributable to resistant infections^3^. AMR is now among the top causes of death globally, exceeding mortality from HIV/AIDS and malaria. Among the most concerning pathogens are the ESKAPE group, *Enterococcus faecium*, *Staphylococcus aureus*, *Klebsiella pneumoniae*, *Acinetobacter baumannii*, *Pseudomonas aeruginosa*, and *Enterobacter* species, responsible for over 100,000 deaths annually^3^. These bacteria have evolved multidrug-resistant (MDR) forms, including carbapenem-resistant *K. pneumoniae* (KPC), methicillin-resistant *S. aureus* (MRSA), and vancomycin-resistant *Enterococcus* (VRE), for which effective treatment options are increasingly scarce^4,5^. In clinical settings, polymicrobial infections are favored primarily due to host-predisposition and inactivation of antibiotics by one MDR pathogen to favor another pathogen. This growing resistance threat demands urgent development of broad-spectrum novel antimicrobial strategies.

Recent advances have demonstrated that bacterial metabolic state profoundly influences antibiotic susceptibility, establishing a new paradigm for adjuvant development. Exogenous metabolites including glucose, pyruvate, glutamine, and formate have been shown to potentiate antibiotic activity against multidrug-resistant and carbapenem-resistant pathogens by stimulating cellular respiration and increasing the proton-motive force (PMF), which indirectly facilitates drug uptake^6–9^. These approaches have demonstrated efficacy across multiple antibiotic classes including aminoglycosides, β-lactams, quinolones, and tetracyclines^6^, with particularly robust effects observed for aminoglycosides whose uptake mechanism is uniquely PMF-dependent^9^. While demonstrating the therapeutic potential of metabolism-targeted strategies, these approaches share several limitations: they rely on native metabolites that are subject to rapid catabolism, producing transient rather than sustained effects, and function through non-specific bioenergetic modulation rather than targeted activation of dedicated transport systems^7,8^. These constraints highlight the need for approaches that provide sustained, transporter-specific activation applicable across diverse antibiotic classes.

**Fig. 1:**
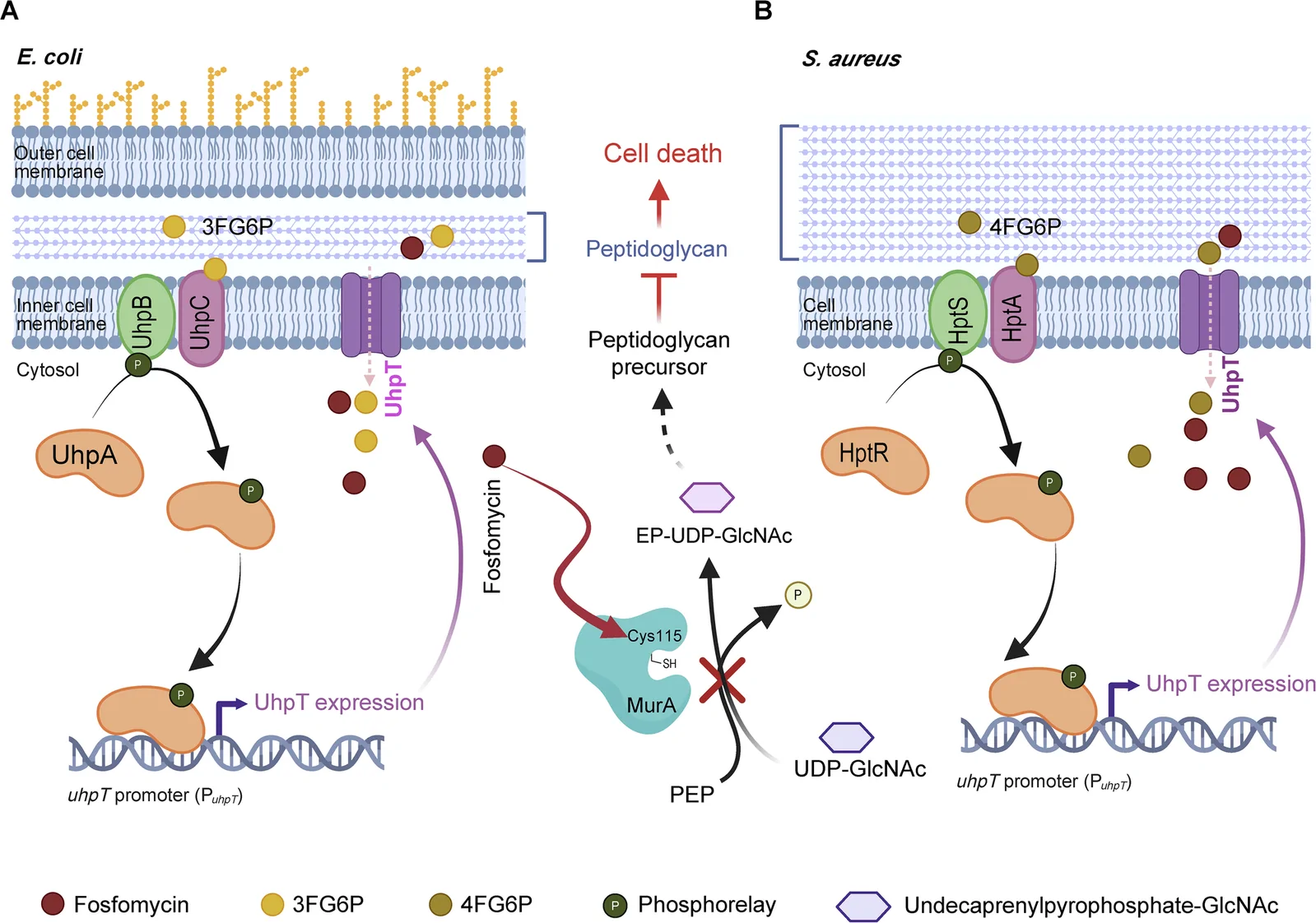
Three-component regulatory system controlling *uhpT* expression in *E. coli* and *S. aureus.* Schematic representation of the regulatory cascade governing *uhpT* induction in (**A**) *E. coli* and (**B**) *S. aureus*. In both organisms, expression is regulated by a three-component system involving the signal transducer UhpC/HptA, the sensor kinase UhpB/HptS, and the response regulator UhpA/HptR, which together mediate transcriptional activation of *uhpT* in response to extracellular glucose-6-phosphate. Figure 1 created in BioRender.

Fosfomycin is a broad-spectrum, bactericidal antibiotic active against both Gram-positive and Gram-negative bacteria. It enters bacterial cells primarily through two conserved transporters: the glycerol-3-phosphate (GlpT) and glucose-6-phosphate (UhpT) transport systems (Fig. 1A, B). Fosfomycin’s unique mechanism, irreversible inhibition of MurA, the enzyme catalyzing the first committed step in peptidoglycan synthesis, provides synergistic activity with β-lactams, aminoglycosides, and fluoroquinolones against MDR ESKAPE pathogens. Clinically, fosfomycin is well tolerated, exhibits favorable pharmacokinetics with broad tissue distribution^10^, and is FDA-approved for oral intake for treating uncomplicated urinary tract infections (UTIs)^11^. Despite these advantages, its clinical utility beyond UTIs remains limited. Oral administration results in low bioavailability (30–37%) and suboptimal distribution to non-urinary tissues, and its relatively higher minimum inhibitory concentration (8–32 mg/L for Enterobacteriaceae) poses additional challenges^12–15^. Resistance can arise *via* mutations in its transporters, GlpT and UhpT, which reduce uptake, or through acquisition of *fosA*, *fosB* or *fosX* gene, which encodes fosfomycin-inactivating enzymes (FosA, FosB, FosX) responsible for fosfomycin resistance by catalyzing the addition of a glutathione, a bacillithiol or a water molecule to the antibiotic^16–18^. While transporter mutations are the more common cause, they confer fitness costs that limit their prevalence, and resistance rates in clinical *Escherichia coli* UTI isolates remain low (~0.7–1.5%)^17–21^. In contrast, *fos* genes encoding fosfomycin-inactivating enzymes are increasingly associated with MDR pathogens, and plasmids co-harboring carbapenemases and *fos* genes raise concerns for rapid dissemination of high-level resistance^22–24^.

Among the known fosfomycin uptake pathways, UhpT is uniquely regulated by three-component signaling cascades—UhpABC in *Escherichia coli* and HptRSA in *Staphylococcus aureus*. In both species, the membrane sensors UhpC (in *E. coli*) or HptA (in *S. aureus*) detects extracellular glucose-6-phosphate (G6P) and activate downstream two-component kinase–response regulator pairs (UhpB–UhpA or HptS–HptR), which in turn drive *uhpT* transcription^25–28^. As a result, external G6P induces UhpT expression and enhances fosfomycin uptake, an effect that has been demonstrated experimentally and is exploited in routine susceptibility testing^29–33^. However, this induction is transient, as G6P is rapidly metabolized by both bacteria and host cells^34^. Thus, strategies to activate this signaling pathway with stable, non-metabolizable inducers could sustain UhpT expression, increase intracellular fosfomycin concentrations, which concomitantly potentiate antibiotic efficacy and limit the emergence of resistant mutants.

Fluorination of carbohydrates may provide the molecules with resistance to enzymatic degradation and improved binding affinity to protein receptors without major structural distortion^35–38^. For example, fluorinated glucose analogues have shown increased transporter-mediated uptake in mammalian cells and enhanced binding to bacterial enzymes^39^. These properties make fluorinated hexose phosphates attractive candidates as metabolically stable inducers for sustained expression of UhpT.

In this study, building upon the metabolic potentiation paradigm, we introduce a distinct strategy that addresses the limitations of current metabolite-based approaches: the use of metabolically stable, synthetic signaling molecules to achieve sustained, direct activation of dedicated antibiotic transport systems. Specifically, we exploit the UhpT transporter regulatory system in *E. coli* and *S. aureus* by employing metabolically resistant fluorinated glucose-6-phosphate analogues (FG6Ps). Two FG6Ps display superior chemical stability and sustained activation of UhpT compared to native G6P, leading to markedly increased influx of fosfomycin. Our data demonstrate that 3-fluoro-D-glucopyranosyl-6-phosphate (3FG6P) in *E. coli* and 4-fluoro-D-glucopyranosyl-6-phosphate (4FG6P) in *S. aureus* potentiate fosfomycin efficacy both in vitro and in vivo, reinstating susceptibility even in clinical isolates harboring a resistance gene, *fosA* in *E. coli* and *fosB* in *S. aureus*. This influx-targeting approach not only expands the therapeutic potential of fosfomycin but also reinforces a new paradigm in antimicrobial adjuvant development, with implications for combating multidrug-resistant ESKAPE pathogens.

## Results

### Chemical synthesis of fluorinated hexose phosphate analogues

To assess the potential for stable induction of UhpT, we generated two FG6Ps, fluorinated at each of 3’- and 4’-carbon replacing the hydroxyl group in the glucose moiety using a known method^40,41^ (Supplementary Fig. S1). Each fluorinated glucose was purchased, and each analogue was enzymatically phosphorylated at its 6’-OH using hexokinase and ATP, followed by purification using anion exchange HPLC. Commercial G6P was included as a reference standard in all subsequent assays.

### Comparative evaluation of UhpT gene expression induced by FG6Ps versus G6P in *E. coli* using fluorescent protein-based reporter constructs

**Fig. 2:**
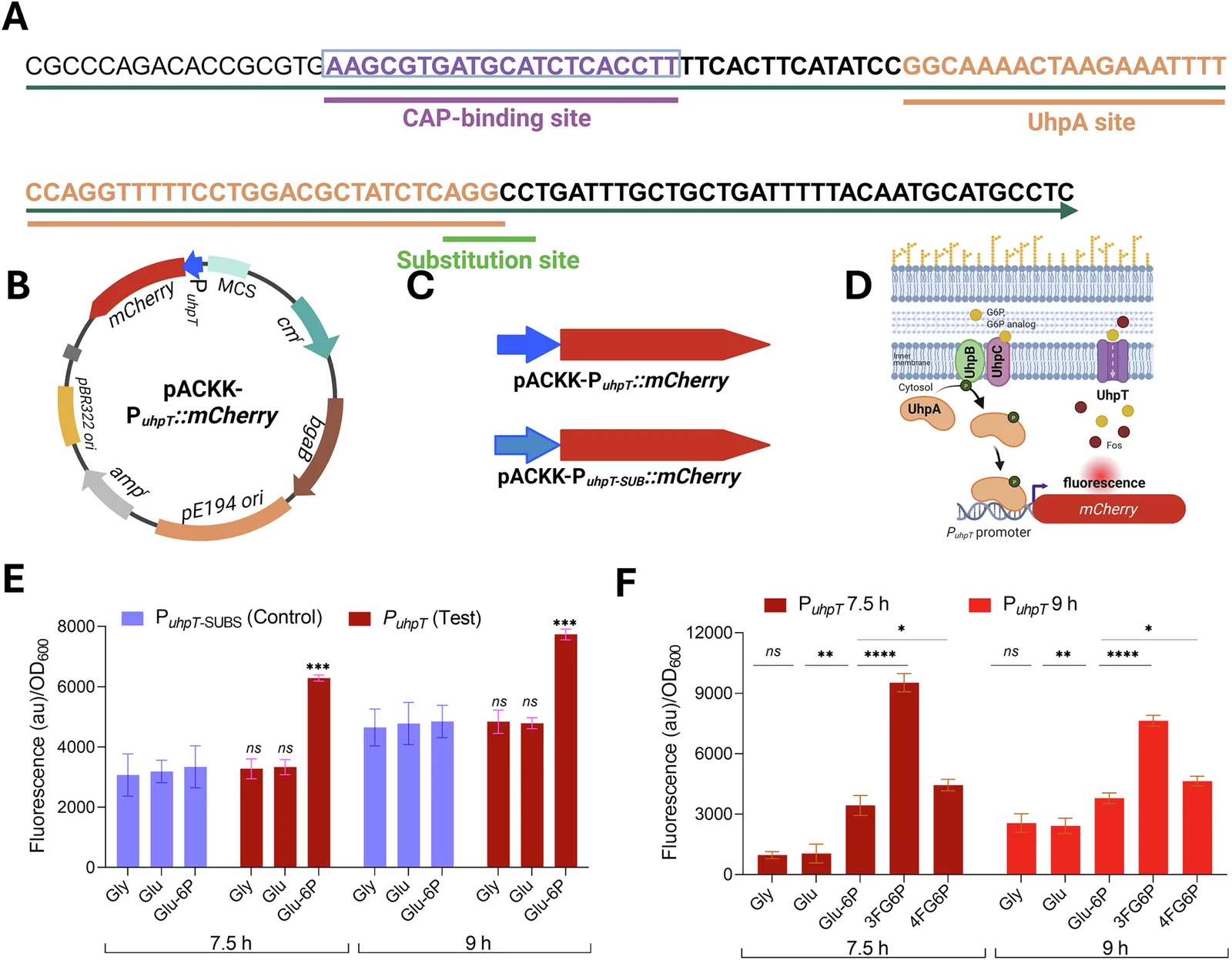
Fluorescent reporter assay for monitoring UhpT induction in *E. coli.* **A** Nucleotide sequence *E. coli* P_*uhpT*_ promoter showing CAP-binding site (cyclic adenosine monophosphate, cAMP), UhpA binding site and the P_*uhpT*_ promoter substitution site to create the control reporter construct with mutated P_*uhpT*_ (P_*uhpT-sub*_), non-responsive to Glucose 6 Phosphate (G6P). **B**–**D** Schematic representation of the pACKK-*mcherry* plasmid (**B**), containing a wild-type P_*UhpT*_ (I) or mutant P_*uhpT-sub*_ (II) promoter (**C**) driving expression of the red-fluorescent protein, mCherry. **D** Schematic diagram showing the test of the reporter strains using Glucose, Glucose 6 Phosphate (G6P) and its fluorinated adjuvants (3FG6P and 4FG6P) in M9 media wherein glycerol was used as a carbon source. **E** Validation of reporter platforms harboring wild-type P_*UhpT*_ or mutant P_*uhpT-sub*_ promoter using fluorometric assay of red-fluorescent mCherry protein as a proxy for *uhpT* transcriptional activity in live *E. coli* cultures. **F** Induction of *uhpT* expression in *E. coli* in response to mono-fluorinated glucose-6-phosphate analogues (3FG6P and 4FG6P), relative to G6P. The mCherry fluorescence was measured in relative fluorescence units (RFU) over time, and values represent the mean ± s.d. of three independent experiments. Statistical significance was calculated by Student’s *t* test (*ns*, not significant; *p < 0.05; **p < 0.01; ***p < 0.001, and ****p < 0.0001). **B**–**D** created in BioRender.

To investigate the induction of *uhpT* expression by G6P and its fluorinated analogues, an engineered red-fluorescent reporter construct was used wherein the *E. coli uhpT* promoter (P_*uhpT*_)^42^ was transcriptionally fused to a promoter-less *mcherry* gene (Fig. 2A–C). The mCherry red-fluorescence under P_*uhpT*_ promoter served as a real-time proxy for *uhpT* activation in live cells while a substitution mutant P_*uhpT-sub*_ acts as control (Fig. 2D, E). Compared with G6P at 50 µM, the same concentration of 3FG6P elicited markedly stronger red-fluorescent signal, indicating enhanced and prolonged P_*uhpT*_ promoter activity while 4FG6P displayed only a slightly enhanced effect (Fig. 2F). These data indicate that fluorination indeed changed the behavior of G6P, and FG6Ps activated the signaling system triggering the UhpT channel to a considerably higher level than G6P did. Thus, these compounds underwent further tests.

### FG6Ps exhibit unique uptake profile in *E. coli* compared to native G6P and enhance fosfomycin uptake

**Fig. 3:**
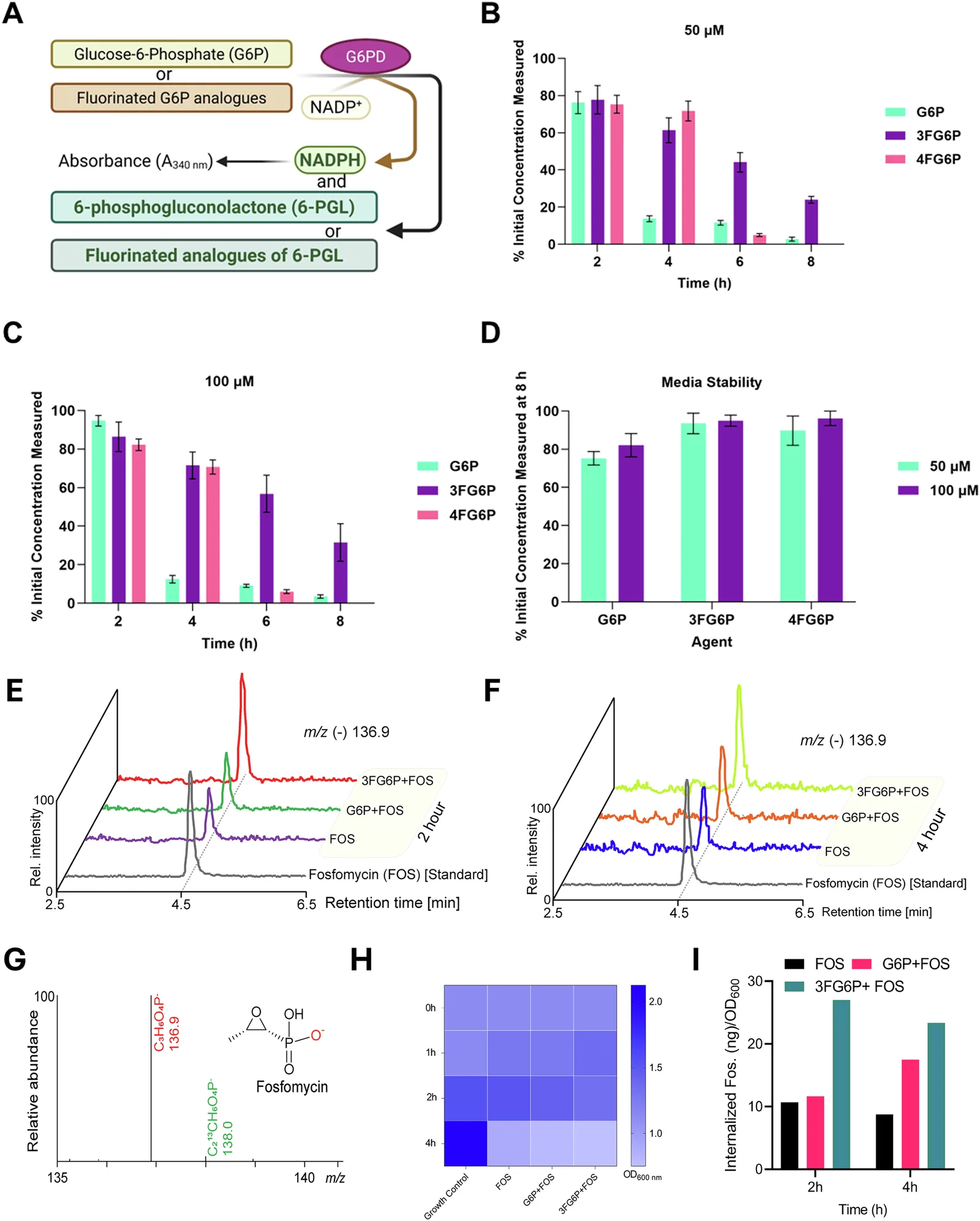
Uptake of G6P and FG6Ps by *E. coli* and their stability in culture medium and the influx of fosfomycin in the absence and presence of G6P or 3FG6P. **A** Schematic diagram showing the modified assay to assess the uptake kinetics of G6P and fluorinated analogues by *E. coli* cells. *Created in BioRender*. **B**, **C** Bar graphs showing the extracellular concentrations of glucose-6-phosphate (G6P), 3-fluoro-G6P (3FG6P), and 4-fluoro-G6P (4FG6P) at defined time points following incubation with *E. coli.* Uptake was quantified using a spectrophotometric assay based on NADPH production via glucose-6-phosphate dehydrogenase (G6PD), with absorbance measured at 340 nm. Assays were performed with an initial substrate concentration of 50 µM (**B**); assays performed with an initial concentration of 100 µM (**C**). Results represent mean ± s.d. from three biological replicates. G6P showed rapid uptake after 4 h, while 3FG6P exhibited delayed and incomplete uptake. 4FG6P demonstrated a distinct profile, with minimal change at 4 h and near-complete depletion by 6 h. **D** Bar graphs showing the concentrations of G6P, 3FG6P, and 4FG6P in LB medium incubated at 37 °C for up to 8 h in the absence of *E. coli*. Compounds were tested at initial concentrations of 50 µM and 100 µM. Measurements were taken at 0 and 6 h by estimating NADPH production by G6PD. All fluorinated analogues showed minimal degradation over time, indicating high chemical stability under these conditions. A ~25% decrease in G6P levels was observed, suggesting enhanced stability conferred by fluorination. Data represent mean ± s.d. of three independent experiments. **E**–**G** A direct time-dependent comparative cell suspension assay for fosfomycin uptake in absence or presence of G6P or 3FG6P using Liquid Chromatography-Mass Spectrometry (LC-MS) analyses, showing chromatograms at 2 h (**E**), 4 h (**F**), and its mass spectrum displaying it *m*/*z* (−) 136.9 (**G**). **H** The growth profile of cell suspension of *E. coli* NCTC12923 (ATCC8739) during 0‒4 h of fosfomycin uptake assay without or with fosfomycin (8 µg/mL) supplemented with 50 µM G6P or 3FG6P. **I** Comparative fosfomycin content per unit OD_600 nm_ (1 × 10^8^ cells) showed the highest level of intracellular fosfomycin supplemented with 3FG6P.

To determine whether differences in uptake of FG6Ps contributed to the observed *uhpT* expression patterns in *E. coli*, we employed a modified version of a previously described colorimetric assay^43,44^, based on the oxidation of G6P by glucose-6-phosphate dehydrogenase in the presence of NADP⁺. The resulting production of NADPH was quantified spectrophotometrically by measuring absorbance at 340 nm, enabling the determination of residual extracellular concentrations of G6P, 3FG6P, and 4FG6P after induction experiments (Fig. 3A). Initial substrate concentrations of 50 µM and 100 µM were used, and at defined time points, the remaining concentration of G6P or fluorinated analogues in the media was measured to assess uptake kinetics (Fig. 3B, C). To verify that fluorination of G6P does not confound these measurements, we determined enzyme kinetics with G6PDH using G6P, 3FG6P, and 4FG6P as substrates (Supplementary Fig. S2), which showed only modest differences in V_max_ with similar K_m_ values. Furthermore, we constructed a standard curve for each substrate in every individual measurement to ensure that the measured concentrations accurately reflected the residual extracellular G6P, 3FG6P, and 4FG6P, respectively.

During the first 2 h, uptake of G6P and FG6Ps at 50 µM was minimal, consistent with early catabolite repression of *uhpT*. From 4 h onward, extracellular G6P decreased sharply, indicating rapid transport or metabolism. By 6 h, G6P levels plateaued at near-undetectable concentrations, likely reflecting assay limits or feedback-mediated downregulation of UhpT (Fig. 3B). In contrast, 3FG6P and 4FG6P showed markedly slower uptake (Fig. 3B). For 3FG6P, extracellular levels decreased only ~10% by 4 h and ~30% by 6 h, with 20–30% still remaining at 8 h, correlating with its sustained induction of *uhpT* (Fig. 3B). The 4FG6P profile is different: concentrations remained stable to 4 h but dropped rapidly by 6 h, becoming nearly undetectable (Fig. 3B). This may explain its modest induction relative to 3FG6P. The uptake profile is similar at 100 µM of each hexose-6-phosphate (Fig. 3C). Together, these data suggest that 3FG6P has a lower uptake kinetics than G6P yet provided a superior level of inducibility to *uhpT* system presumably due to higher metabolic-stability of fluorinated glucose analogues in *E. coli*. Next, we checked the stability of 3FG6P and 4FG6P before using it as potentiator molecule to enhance the fosfomycin influx.

The stability of these compounds in LB medium at 37 °C was assessed to determine whether observed decreases in extracellular concentration were attributable to bacterial uptake rather than chemical degradation. As shown in Fig. 3D, absorbance measurements at 0 and 8 h for 50 µM and 100 µM of each compound revealed minimal changes, indicating that reductions observed in the presence of *E. coli* result predominantly from cellular uptake. Notably, G6P exhibited a slight decrease in stability compared to FG6Ps, suggesting that fluorination may confer enhanced chemical stability in this medium—a phenomenon consistent with the known stabilizing effects of hydroxyl group substitution by fluorine^45–47^. Together, these results suggest that the substantial increase in the UhpABC signaling can be, at least partially, attributed to the prolonged extracellular presence of FG6Ps, which sustains the signaling.

To directly measure fosfomycin uptake, we performed time-course LC-MS analysis of intracellular fosfomycin in *E. coli* ATCC 8739 treated with fosfomycin (8 μg/mL) alone or supplemented with G6P or 3FG6P (50 μM) (Fig. 3E–I, Supplementary Fig. S3). Cell density increased slightly over the first 2 h under all conditions but declined thereafter in the presence of fosfomycin, with 70.7% and 67.1% of initial cell numbers remaining at 4 h for G6P and 3FG6P treatments, respectively (Fig. 3H). Critically, cells treated with fosfomycin plus 3FG6P accumulated over 2.3-fold higher intracellular fosfomycin concentrations (normalized to cell density) compared to fosfomycin alone or fosfomycin plus G6P (Fig. 3I), providing direct evidence that 3FG6P enhances fosfomycin influx.

### FG6Ps potentiate fosfomycin activity in vitro

To determine the functional relevance of FG6P-dependent reporter gene expression, we next measured their effect in potentiating fosfomycin toxicity in bacteria. We tested on a panel of *E. coli* strains, including DH5α, and NCTC12923. The latter strain is resistant to gentamicin, amikacin and tobramycin. The MICs of fosfomycin under each condition are shown in Table 1.

**Table 1:** Minimum inhibitory concentrations (MICs) of fosfomycin in the presence or absence of G6P analogues

Agent	MIC (µg/mL)				
	*E. coli* DH5α	*E. coli* NCTC12923	*E. coli A004 harboring fosA*	*S. aureus* USA300	*S. aureus COL harboring fosB*
Fosfomycin	8	16	ND	8	ND
Fosfomycin + 50 µM G6P	1	2	≥256	1	≥256
Fosfomycin + 50 µM 3FG6P	0.125–0.25	0.25–0.5	2	1	ND
Fosfomycin + 50 µM 4FG6P	0.125–0.25	0.5	ND	0.25	8
3FG6P	>256	>256	ND	>256	ND
4FG6P	>256	>256	ND	>256	ND

*ND* not determined.

Co-administration of G6P enhanced fosfomycin activity by a factor of up to 8 against *E. coli*; however, both fluorinated analogues conferred significantly greater potentiation. 3FG6P and 4FG6P reduced the MIC of fosfomycin by up to 128-fold, indicating a profound enhancement of susceptibility in *E. coli*. It was shown that 3FG6P and 4FG6P did not have an antibacterial activity against both *E. coli* and *S. aureus* (Table 1), which strongly indicates that the substantial reduction in MICs of fosfomycin is indeed caused by the enhanced intake of fosfomycin through action of FG6Ps. Then, we tested the fosfomycin potentiation effect of 3FG6P against a clinical UTI isolate harboring *fosA* gene, which encodes a fosfomycin inactivating enzyme and confers fosfomycin resistance^48^. MIC of fosfomycin for this strain is ≥256 µg/mL^48^, but when 50 µM of 3FG6P was present instead of G6P, the MIC dramatically dropped to 2 µg/mL. This clearly indicates the fosfomycin influx was enhanced sufficiently to overwhelm the action of the FosA enzyme mediated inactivation of fosfomycin. These findings reveal clear fosfomycin potentiation effect of FG6Ps *via* the signaling system triggering the UhpT expression.

#### Enhanced bactericidal activity of fosfomycin by FG6Ps in *E. coli*

**Fig. 4:**
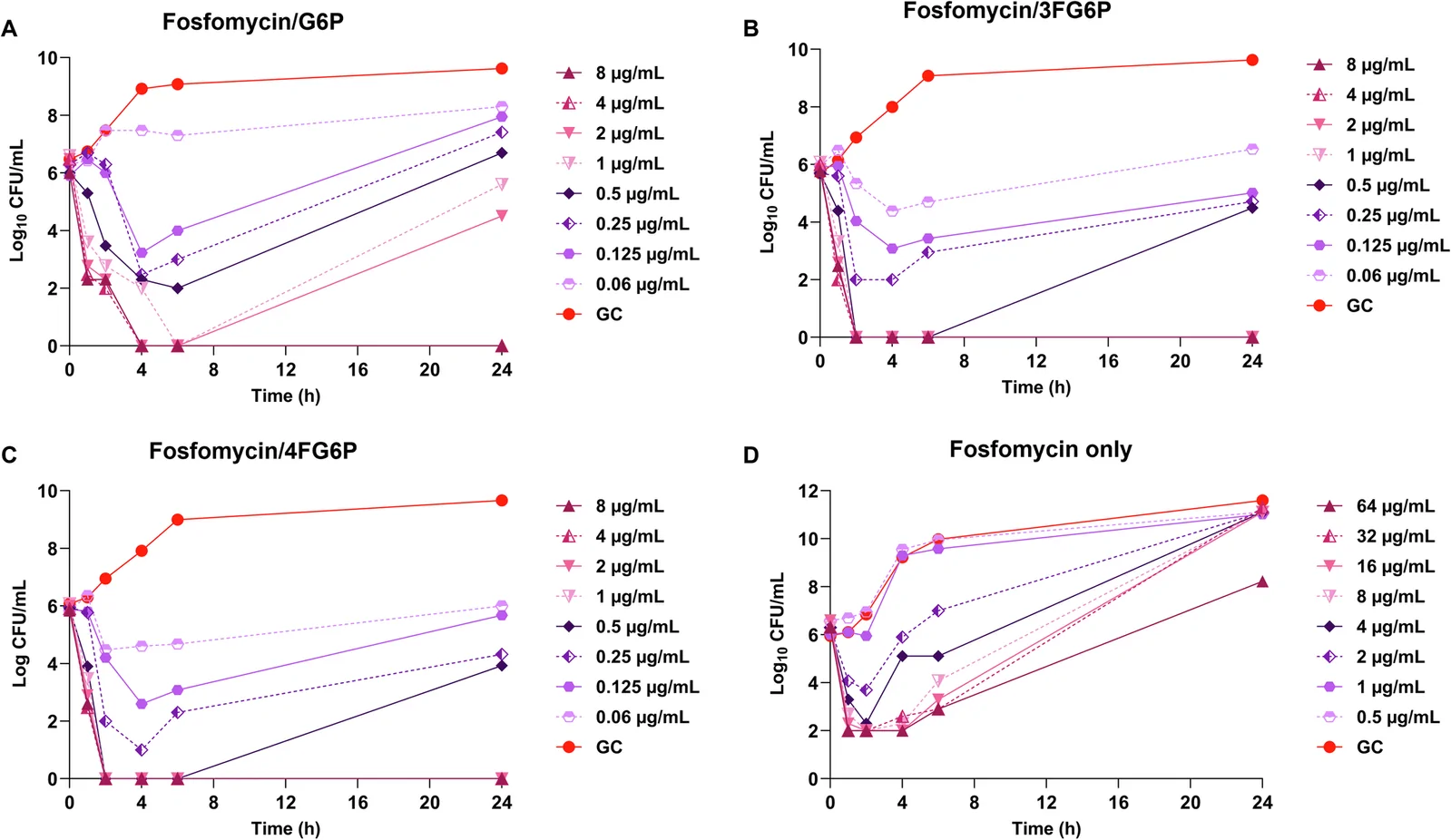
Time-kill kinetics of fosfomycin in combination with glucose-6-phosphate (G6P) and fluorinated glucose-6-phosphate analogues (FG6Ps) against *E. coli*. Bacterial killing was assessed over 24 h using time-kill assays with *E. coli* in the presence of 50 µM of G6P (**A**), 3FG6P (**B**) or 4FG6P (**C**), or fosfomycin alone (**D**) and increasing concentrations of fosfomycin. Data represents CFU/mL from biological replicates measured at defined time points. Enhanced bactericidal activity and suppression of regrowth were observed with fluorinated analogues, particularly 3FG6P.

Time-kill assays were conducted to evaluate the bactericidal activity of fosfomycin in combination with G6P and FG6Ps at a concentration of 50 µM against *E. coli* DH5α. Figure 4 presents representative time-kill curves illustrating the effect of G6P, 3FG6P, and 4FG6P across a range of fosfomycin concentrations.

Fosfomycin combined with 50 µM G6P exhibited concentration-dependent bactericidal activity against *E. coli* DH5α over an 8-h period (Fig. 4A). At fosfomycin concentrations of 0.25–8 µg/mL, a >3-log reduction in viable bacterial counts was observed within 4 h, demonstrating potent killing; however, partial regrowth occurred at 2 μg/mL and below after 24 h, indicating incomplete eradication at lower concentrations. Higher concentrations maintained sustained suppression throughout the 24-h period.

In contrast, in the presence of 50 µM 3FG6P (Fig. 4B), fosfomycin elicited more rapid bactericidal activity, achieving >3-log reductions within 2 h across a broader concentration range (0.125–8 µg/mL). Partial regrowth was noted at 0.5 µg/mL and below after 24 h, whereas higher concentrations maintained prolonged bacterial suppression. Similarly, 4FG6P enhanced killing at 0.125–8 µg/mL within 2 h, with regrowth observed at 0.5 µg/mL and lower concentrations during extended incubation (Fig. 4C). These data demonstrate the superior and accelerated bactericidal potentiation of fosfomycin by FG6Ps against *E. coli* relative to G6P.

For comparison, we have also conducted time-kill assays for fosfomycin alone without G6P or FG6P (0.5–64 μg/mL). As observed with the presence of G6P or FGPs, rapid bactericidal activity was achieved within 2 h, but across a narrower concentration range (4–64 μg/mL). This effect was also not sustained, with recovery of viable cells evident by 6 h, and by 24 h bacterial growth approached those observed for the untreated control. What’s more, complete clearance of bacterial cells was not achieved under any condition, with maximal killing limited to approximately 4 log_10_ reduction in CFU/mL.

### 3FG6P suppresses the emergence of fosfomycin resistance

**Fig. 5:**
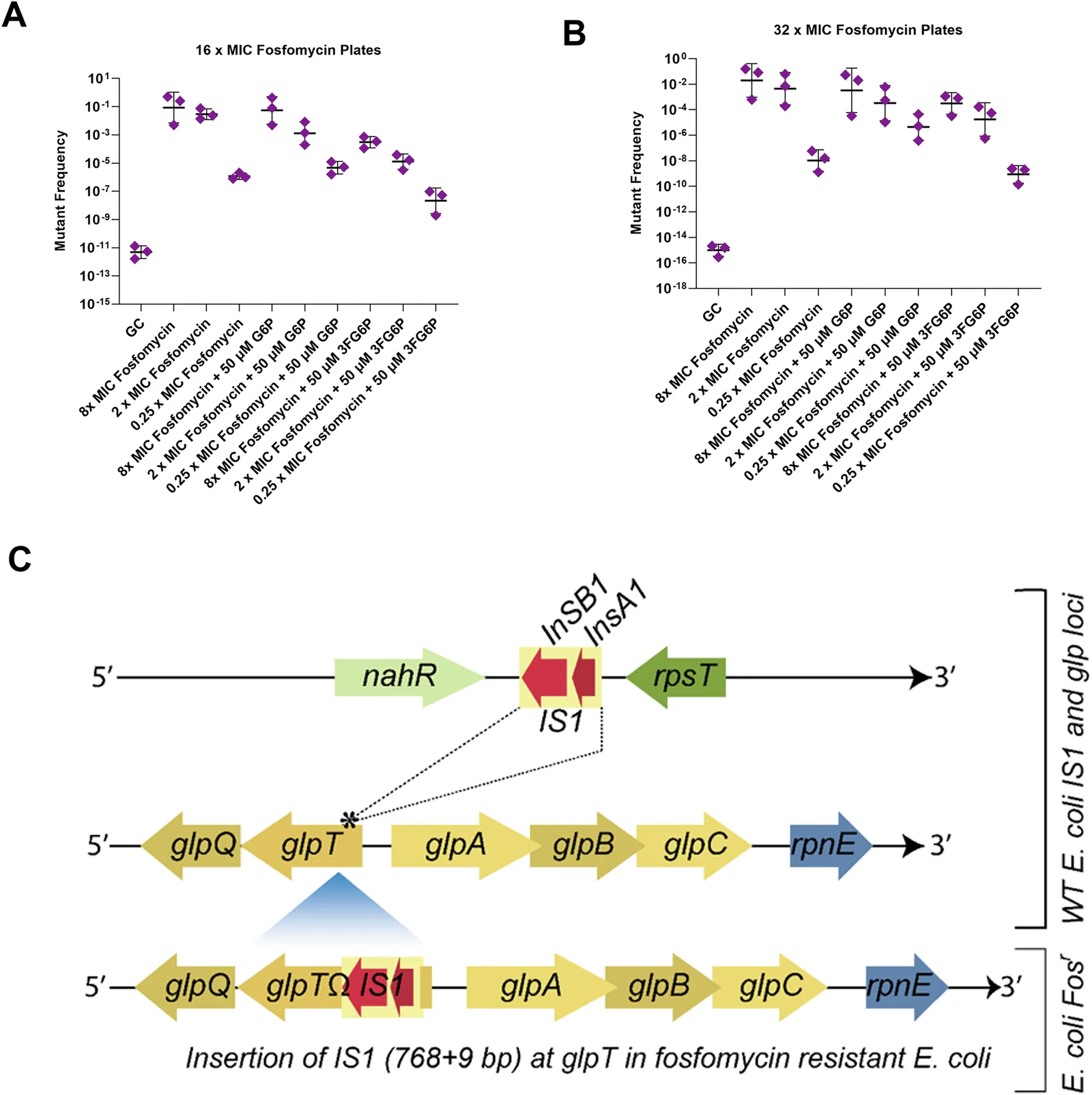
Emergence of fosfomycin resistance in *E. coli* DH5α. **A**, **B** Mutational frequencies after 7-day serial passage with fosfomycin ± metabolic adjuvants (16× and 32× MICs). Fosfomycin alone caused a strong, concentration-dependent increase in resistance, glucose-6-phosphate (G6P) moderately reduced emergence, and 3-fluoro-glucose-6-phophate (3FG6P) profoundly suppressed resistance, approaching baseline levels. Data show mean ± s.d. of three biological replicates; individual replicates are plotted to illustrate variability and consistent trends. **C** The repeated 7-day serial passage of *E. coli* DH5α with fosfomycin at 8 µg/mL (8× MICs) of fosfomycin and 50 µM of metabolic adjuvants (G6P or 3FG6P) showed the emergence of fosfomycin-resistant *E. coli* DH5α. PCR amplification of *murA*, *uhpA*, *uhpB*, *uhpC*, *uhpT*, and *glpT* gene showed insertion of *IS1* element in *glpT* gene. The insertion of *IS1* element resulted in truncated 68 amino acid GlpT, which could be plausible cause fosfomycin resistance in adaptively evolved strains on fosfomycin.

To examine the impact of 3FG6P on fosfomycin resistance evolution, *E. coli* DH5α populations were serially passaged for 7 days in growth medium alone, fosfomycin at 8×, 2×, or 0.25× MIC, or fosfomycin supplemented with 50 µM G6P or 50 µM 3FG6P. Cultures were plated on fosfomycin-containing agar (16× and 32× MIC) at day 7, and mutational frequencies were determined (Fig. 5).

Exposure to fosfomycin alone led to a marked, concentration-dependent increase in mutational frequency compared to growth controls, ranging from 1.29 (± 0.71) × 10⁻⁶ at 0.25× MIC to 2.57 ( ± 2.53) × 10^−1^ on 16× MIC plates, and from 2.50 ( ± 2.97) × 10⁻⁸ to 7.99 ( ± 7.82) × 10⁻² on 32× MIC plates, consistent with strong selective pressure favoring resistant variants. Supplementation with G6P moderately reduced resistance emergence, with mutational frequencies generally 10- to 100-fold lower than fosfomycin alone (e.g., 3.29 ( ± 4.35) × 10⁻³ at 2× MIC on 16× MIC plates), reflecting its role in enhancing fosfomycin uptake via the UhpT transporter. Remarkably, supplementation with 3FG6P profoundly suppressed resistance across all tested concentrations, with frequencies approaching baseline levels (5.01 ( ± 4.67) × 10⁻⁸ to 3.93 ( ± 3.07) × 10⁻⁴), even at high fosfomycin exposure. Despite significant suppression of resistance by 3FG6P (Fig. 5A, B), resistant variants still emerged at low frequencies. To investigate the genetic basis of this residual resistance, we performed targeted sequencing of key resistance determinants (*uhpT*, *uhpABC*, *glpT*, *murA*) in three independent isolates selected with fosfomycin plus G6P or 3FG6P. No mutations were detected in *uhpT, uhpABC*, or *murA*. However, all fosfomycin-exposed isolates harbored a 777 bp insertion in *glpT*, consisting of the IS1 transposable element (768 bp) plus a 9 bp target site duplication (Supplementary Fig. S4A–C, Fig. 5C). This insertion truncates GlpT to 68 amino acids, disrupting the alternative glycerol-3-phosphate transporter pathway for fosfomycin uptake.

Mutational frequencies measured on 16× and 32× MIC plates are summarized in Supplementary Table S3, highlighting the consistent trends across biological replicates. Some variability among replicates was observed, particularly at higher fosfomycin concentrations, indicating heterogeneous mutational responses under strong selective pressure.

#### 3FG6P activates the UhpT regulatory pathway in *E. coli*

The assessment of transcriptional response towards FG6Ps at the mRNA level is essential before using in vivo validation of these adjuvants as potentiator molecules. To this end, we selected 3FG6P with G6P as a control to perform quantitative reverse-transcription polymerase chain reaction (qRT–PCR) analysis to evaluate the expression of *uhpA*, *uhpB*, *uhpC*, and *uhpT* in *E. coli* NCTC12923 in the presence of the compound.

In M9 minimal medium supplemented with glycerol (M9-Gly), treatment with 3FG6P led to marked upregulation of all four genes, with fold changes of 4.2 (*uhpA*), 6.0 (*uhpB*), 6.9 (*uhpC*), and 46.4 (*uhpT*) relative to untreated controls (Supplementary Fig. S5A). These levels were consistently higher than those induced by G6P, which elicited fold changes of 1.3, 2.8, 11.7, and 32.2, respectively. Notably, the greatest induction was observed for *uhpT*, consistent with its role as the primary fosfomycin transporter.

In a more nutrient-rich condition (M9 medium supplemented with both glycerol and glucose), a similar transcriptional profile was observed, with 3FG6P inducing *uhpA*, *uhpB*, *uhpC*, and *uhpT* by 49.0-, 54.2-, 1.9-, and 22.2-fold, respectively (Supplementary Fig. S5B). Again, expression levels of *uhpA*, *uhpB*, and *uhpT* in response to 3FG6P were significantly elevated compared to G6P.

These results indicate that 3FG6P robustly activates not only the *uhpT* gene but also its upstream transcriptional regulators *uhpA* and *uhpB*. This coordinated upregulation suggests a mechanism by which 3FG6P may enhance fosfomycin susceptibility, by increasing the expression of key components of the UhpT uptake pathway and thereby facilitating greater intracellular accumulation of the antibiotic.

### Fosfomycin efficacy is markedly enhanced in vivo by co-administration with 3FG6P in the *Galleria mellonella* larvae infection model

**Fig. 6:**
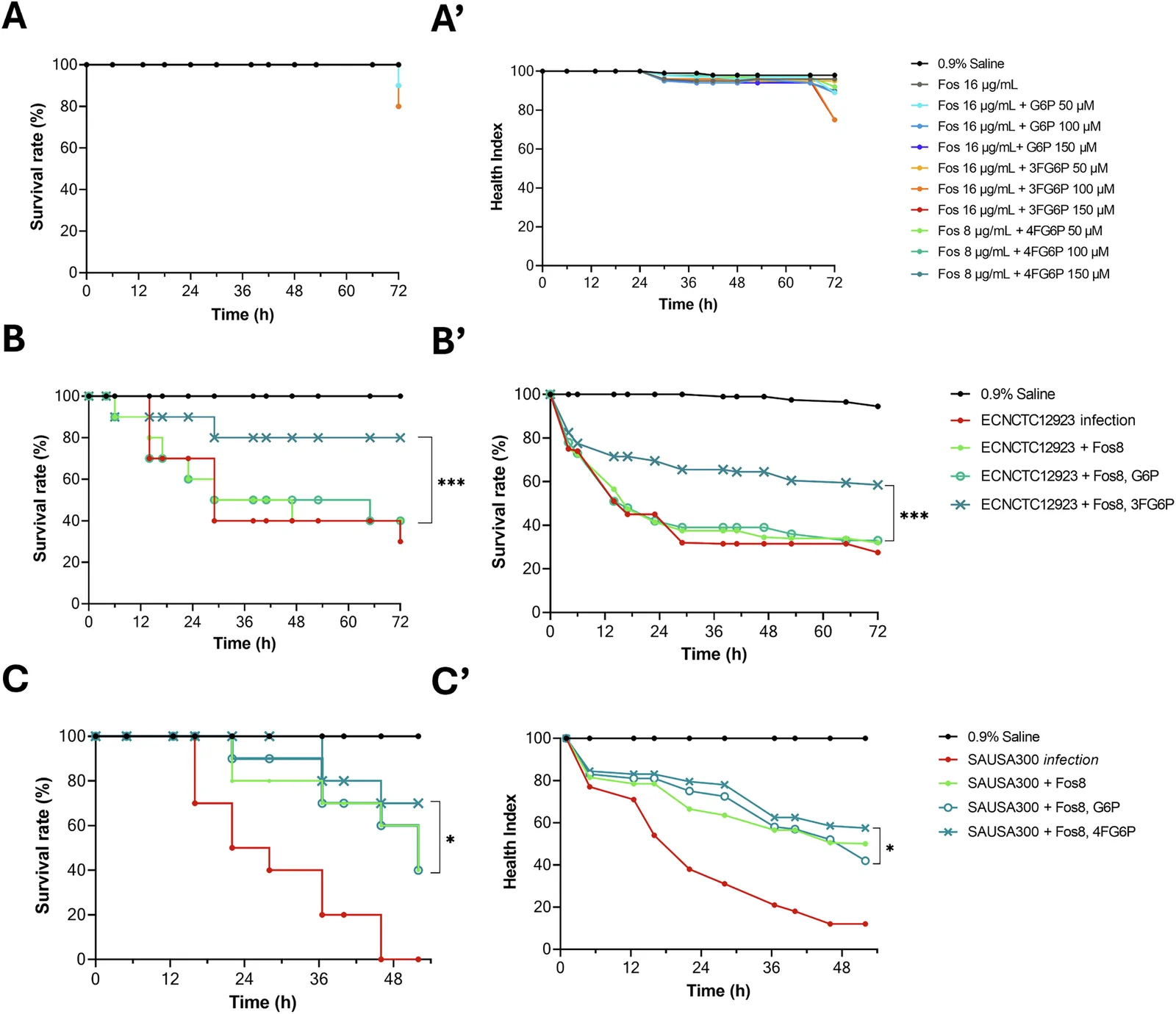
In vivo potentiation of fosfomycin by fluorinated glucose-6-phosphate analogues in *Galleria mellonella* infection models. Establishment of *G. mellonella* larvae infection model to assess their survival and protection against *E. coli* NCTC12923 or *S. aureus* USA300 infections by assessing the health-index (*n* = 10 per group). The larvae were infected with established bacterial inoculants (CFUs), and treated with saline (control), fosfomycin alone or fosfomycin supplemented with G6P or its mono-fluorinated analogues. Larval survival and heath-index were monitored over 72 h following injection with increasing bacterial inoculants. Larvae were incubated at 37 °C and monitored for survival, motility, cocoon formation, and melanization over a 72-h period to calculate their health-index with time. **A**–**A’** Survival (**A**), and toxicity assessment in terms of health-index (**A’**) of fosfomycin alone or in combination with glucose-6-phosphate (G6P) or 3-fluoro-glucose-6-phosphate (3FG6P) in *G. mellonella* larvae. No significant toxicity was observed up to 66 h post-injection. **B**–**B’** Survival of *G. mellonella* (**B**), and their protection against *E. coli* NCTC12923 infection by assessing health-index (**B’**). **C**–**C’** Survival of *G. mellonella* (**C**), and health-index (**C’**) of larvae infected with 20 µL of *S. aureus* USA300, and treated with saline (control), fosfomycin alone (8 µg/mL for *E. coli*; 16 µg/mL for *S. aureus*), or in combination with 50 µM G6P or 4FG6P. The fluorinated 3FG6P and 4FG6P analogues showed better potentiation of fosfomycin than G6P against *E. coli* NCTC12923 or *S. aureus* USA300 infections, respectively. Data are presented as the means ± standard deviations of the means. Statistical comparisons among groups were conducted using one-way ANOVA followed by a post-hoc Tukey’s test. Differences were considered statistically significant for *p* values < 0.05 and labeled in the graph as ns, nonsignificant; *p ≥ 0.05; and **p ≤ 0.01, and ***p < 0.001.

To evaluate the in vivo potentiation of fosfomycin, we employed a *G. mellonella* infection model with *E. coli* using 3FG6P. Prior to infection studies, the toxicity of fosfomycin alone or in combination with G6P or 3FG6P was assessed by injecting 20 µL of each formulation into the left posterior leg of larvae (*n* = 10). Larval survival remained at 100% up to 66 h post-injection, with only a modest decline (10–20%) at 72 h (Fig. 6A), indicating minimal toxicity of the treatments with similar health-index as placebo group (Fig. 6A’). Infection parameters were optimized by inoculating larvae with varying CFUs of *E. coli* NCTC12923 (or *S. aureus* USA300, discussed later) and monitoring survival over 72 h (Supplementary Fig. S6A, B). Saline-treated larvae served as placebo controls. After determination of effective CFUs, wax-worm larvae were administered with bacterial inocula into the left posterior leg, followed by treatment with fosfomycin—with or without 3FG6P—into the right posterior leg to evaluate antibiotic potentiation in vivo (Fig. 6B).

Co-administration of fosfomycin with 3FG6P significantly improved survival in *E. coli*-infected larvae, achieving 80% survival compared to 30%, 40%, and 40% survival in untreated, fosfomycin-only, and fosfomycin plus G6P groups, respectively (Fig. 6B). This clearly demonstrated the substantial potentiation effect of 3FG6P on fosfomycin efficacy against *E. coli* NCTC12923. Beyond survival, larvae treated with fosfomycin in combination with 3FG6P displayed enhanced physical health, including increased motility, reduced melanisation, and consistent cocoon formation, relative to larvae treated with fosfomycin plus G6P (Fig. 6B’). Together, these results demonstrate that fluorinated glucose-6-phosphate analogues potentiate fosfomycin efficacy in vivo—likely via UhpT-mediated uptake—and represent a promising approach to circumvent antibiotic resistance in clinically relevant pathogens.

### 4FG6P potentiates fosfomycin most effectively in *S. aureus*

Encouraged with the data with Gram-negative *E. coli*, we expanded our study to Gram-positive *S. aureus*, which also has a similar UhpT activating system, HptRSA. Thus, we tested the MIC enhancing effect of FG6Ps for *S. aureus* USA300 strain that carries multiple antibiotic resistances including ciprofloxacin, levofloxacin, gentamicin, vancomycin, β-lactams, and chloramphenicol. Similar trends were observed, with the MIC reduced by G6P and FG6Ps by at least 8-fold. Notably, 4FG6P exhibited markedly superior potentiation, even to 3FG6P, reducing the MIC of fosfomycin by up to 32-fold (Table 1). This superior enhancement highlights the potential of 4FG6P as a species-specific adjuvant. Then, we tested 4FG6P as the potentiator against a clinical isolate *S. aureus* COL strain harboring *fosB* gene encoding a fosfomycin-inactivating enzyme, of which MIC is >256 µg/mL^49^. It was shown that the MIC of fosfomycin against this resistant *S. aureus* was 8 µg/mL in presence of 50 µM 4FG6P, displaying a clear resistance reversing effect.

Then, we further tested the potentiating effect of 4FG6P on fosfomycin in the *G. mellonella* infection model with *S. aureus* USA300. Just as in the same model with *E. coli*, treated larvae exhibited 0% survival, fosfomycin alone conferred 60% protection, whereas fosfomycin supplemented with G6P paradoxically reduced survival to 40%, which may indicate the ineffectiveness of G6P. Notably, fosfomycin combined with 4FG6P increased survival to 75%, demonstrating superior protective efficacy (Fig. 6C). The improved physical health was also observed, which showed that larvae treated with fosfomycin in combination with 4FG6P displayed increased motility, reduced melanization, and consistent cocoon formation, relative to larvae treated with fosfomycin plus G6P (Fig. 6C’). For comparison, when we used 3FG6P as a potentiator, it was shown to protect 80% larvae but at higher concentration (16 µg/mL) of fosfomycin, which presumably shows species-specificity (Supplementary Fig. S7A, B). Together, these findings show that FG6Ps are effective potentiators of fosfomycin in bacteria with similar G6P sensitive signaling systems triggering UhpT regardless of Gram-positive or Gram-negative species.

### 4FG6P exhibits an enhanced binding affinity for HptA compared to the native substrate

Given that the expression of UhpT is regulated by two-component signaling systems—UhpBC in *E. coli* and HptRS in *S. aureus*—which are activated by G6P via the respective sensor proteins UhpC and HptA, we investigated the binding affinities of an FG6P to the sensor proteins as a means of assessing the impact of fluorination on signaling activation. In *E. coli*, UhpC is a multi-pass transmembrane protein^27,50^, precluding its use in direct biophysical binding studies due to difficulties in soluble expression and purification. We therefore focused on *S. aureus* HptA, a soluble sugar phosphate-binding protein^28,51^, which allowed us to experimentally assess ligand binding using isothermal titration calorimetry (ITC). Binding affinities of HptA were measured for glucose, 4FG6P and G6P as a reference control. These measurements provide mechanistic insight into how fluorination alters ligand recognition and potentially modulates downstream signaling cascades involved in fosfomycin uptake.

**Fig. 7:**
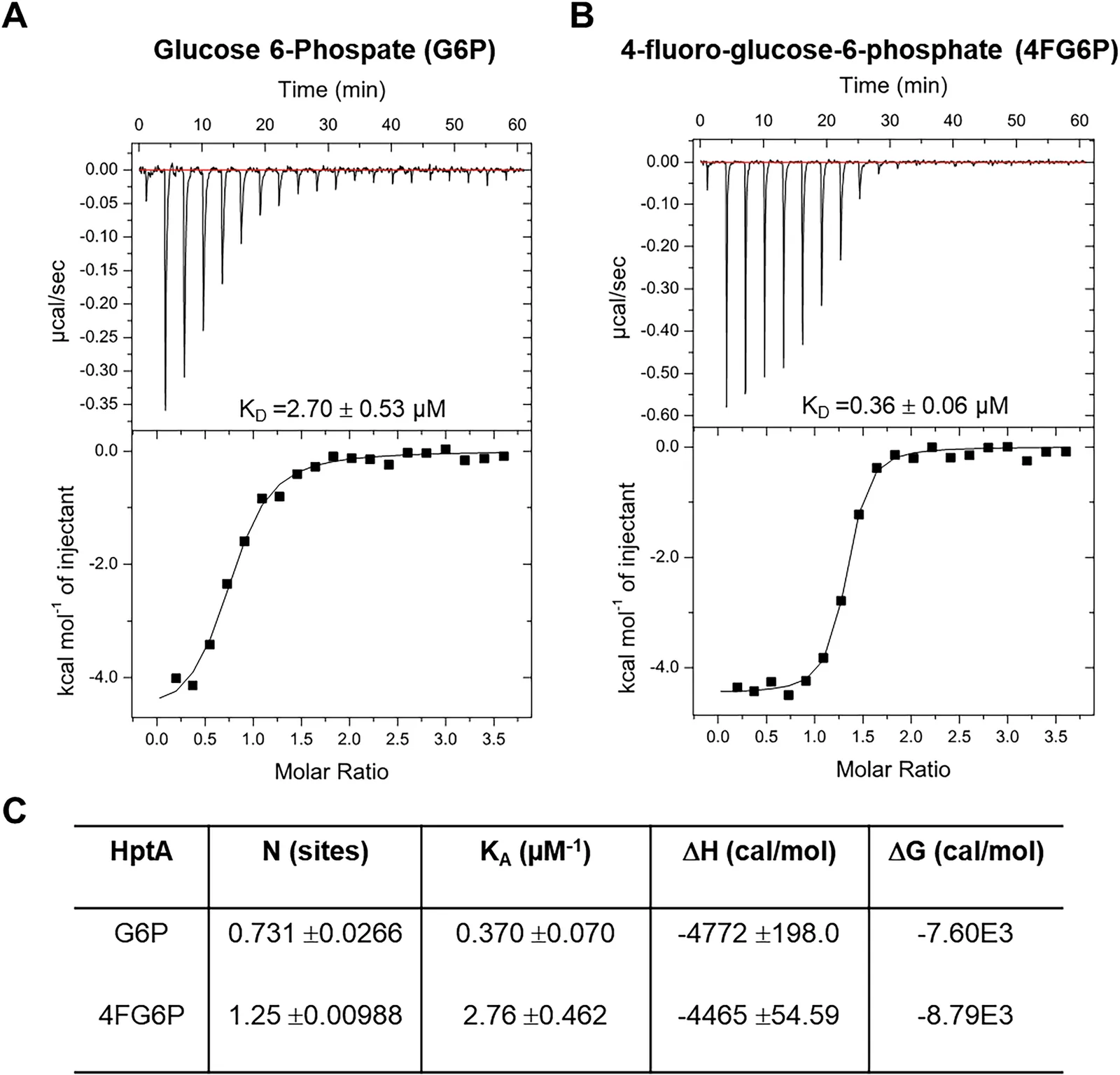
Binding affinity of hexose phosphates to HptA measured by isothermal titration calorimetry (ITC). **A**, **B** Representative ITC thermograms and binding isotherms for the interaction of HptA with G6P (**A**), and 4FG6P (**B**), at 25 °C in 20 mM Tris-HCl, 150 mM NaCl (pH 7.5). HptA bound 4-fluoro-glucose-6-phosphate (4FG6P) with the 7.5 fold higher affinity (K_D_ = 0.36 µM) (**B**) as compared to glucose-6-phoshate (G6P) (K_D_ = 2.70 µM) (**A**), while no binding was observed for glucose. **C** Summary of binding affinities and thermodynamic parameters. Free energy (ΔG) was calculated from enthalpy (ΔH) and entropy (ΔS) values determined from ITC measurements.

ITC revealed that *S. aureus* HptA binds 4FG6P with markedly higher affinity than the native ligand, G6P. The dissociation constant (*K*_*D*_) for 4FG6P was determined to be 0.36 μM, compared to 2.70 μM for G6P, indicating a strong preference for the fluorinated analogue (Fig. 7). The ITC measurement showed a negligible interaction of HptA with glucose (Supplementary Fig. S8A, B). Thermodynamic analysis showed that the trend in binding affinities was mirrored by changes in free energy (ΔG), calculated from enthalpic (ΔH) and entropic (ΔS) contributions, further supporting the enhanced interaction between HptA and 4FG6P. Together, it can be suggested that the higher binding affinity of FG6Ps to the sensor protein may contribute substantially to the observed explosive signaling trigger in combination with their slow uptake (i.e., removal) and longer presence around the sensor protein described earlier.

## Discussion

Fluorinated carbohydrates are known to exhibit resistance to enzymatic degradation and reduced metabolic turnover^38,52^, and the fluorinated version of G6P, an FG6P, is expected to benefit from such characteristics. Subsequently, persistence of FG6P is expected to strengthen the signaling system triggering the expression of UhpT, which will enhance the influx of fosfomycin into bacteria unlike native G6P, expected to be degraded in metabolism. Thus, it was found that FG6Ps could simultaneously evade rapid catabolism and function as potent activators of UhpT expression. Furthermore, we showed, using an mCherry-based fluorescence reporter system in *E. coli*, that fluorinated hexose phosphates, 3FG6P and 4FG6P induce an improved activation of the P_*uhpT*_ promoter compared to native G6P with the former displaying a markedly strong and sustained induction (Fig. 2). This suggests that these analogues not only engage the *uhpT* regulatory pathway more effectively but may also overcome the transient nature of native substrate induction due to rapid metabolism.

Our uptake assays revealed that FG6Ps exhibit distinct uptake kinetics compared to G6P, with slower but more prolonged extracellular depletion. This behavior likely reflects altered transporter affinity or regulatory feedback, consistent with the differential fluorescence induction profiles observed (Figs. 2 and 3). Importantly, chemical stability assays confirmed these compounds remain stable in growth medium, indicating that observed decreases in extracellular levels reflect active bacterial uptake rather than spontaneous degradation. Fluorination thus appears to confer enhanced molecular stability alongside unique uptake dynamics, which may collectively contribute to strong and stable transporter activation. In addition, isothermal titration calorimetry demonstrated enhanced binding affinity of 4FG6P for the *S. aureus* sensor protein HptA compared to native G6P, providing another layer of evidence for superior signaling and transporter activation by FG6Ps. Although direct binding studies for *E. coli* UhpC remain technically challenging, these results suggest that fluorination modifies ligand recognition to promote stronger and more stable activation of the relevant two-component systems. Notably, these data suggest that selected FG6Ps changed ligand recognition in a way that the sensor proteins favor fluorination while transporter UhpT does not. The combination of such contradictory behaviors of the sensors and the transporter likely contributed substantially to the observed stronger enhancement of signaling. Furthermore, qRT–PCR analysis conducted in *E. coli* revealed that 3FG6P strongly upregulates not only *uhpT* but also upstream regulatory genes *uhpA*, *uhpB*, and *uhpC*, indicating activation of the full UhpT regulatory cascade, which may be amplified by the stable and stronger effect of FGPs. This coordinated induction likely facilitates considerably increased transporter abundance and antibiotic uptake. While direct UhpT protein quantification would complement these transcriptional measurements, the strong correlation between promoter activation, transcript levels, and multiple phenotypic outcomes provides convergent functional evidence for UhpT upregulation.

Notably, direct LC-MS quantification demonstrated that 3FG6P increases intracellular fosfomycin accumulation more than 2.3-fold compared to fosfomycin alone or with G6P (Fig. 3I), providing definitive evidence that sustained UhpT activation enhances antibiotic influx. This directly connects promoter activation, transcript upregulation, and the ultimate phenotypic outcome: elevated intracellular drug concentrations that can overwhelm resistance mechanisms.

The potentiation of fosfomycin activity by FG6Ps was striking. While G6P improved fosfomycin efficacy, fluorinated analogues elicited substantially greater reductions in MICs, with both 3FG6P and 4FG6P proving highly effective in *E. coli*. The same trend of fosfomycin efficacy enhancement was also observed in *S. aureus*, demonstrating the same effect of HptRSA signaling on UhpT, but in this case, 4FG6P displayed superior activity. As FG6Ps did not show any antibacterial activity, such fosfomycin efficacy enhancement was the strong indication of the over-expression of fosfomycin transporter UhpT triggered by FG6Ps. Importantly, *E. coli* and *S. aureus* clinical isolates harboring fosfomycin-resistant gene *fos*A (*E. coli*) or *fos*B (*S. aureus*) have become sensitive again in the presence of 3FG6P and 4FG6P, respectively. This is likely attributed to the overwhelming influx of fosfomycin that saturates the Fos-enzymes and creates a sufficient surplus to kill bacteria. This is an important example that the understanding and utilization of an influx mechanism can lead to successful fight against resistance. It is to be noted that differently fluorinated G6P analogs showed a different activity between *E. coli* and *S. aureus*. This divergence likely reflects species-specific differences in the structure and interactions of sensor proteins (i.e., UhpC vs HptA) in the regulation of UhpT and the membrane environment between Gram-negative and Gram-positive bacteria, emphasizing the need for tailored approaches when designing fosfomycin adjuvants.

3FG6P was also found to profoundly suppress the emergence of fosfomycin resistance in *E. coli*. Serial passage experiments revealed that populations exposed to fosfomycin alone developed resistance in a strong, concentration-dependent manner, whereas supplementation with G6P moderately mitigated this effect. Remarkably, 3FG6P maintained mutational frequencies near baseline across all conditions, even at high drug exposure, displaying robust and reproducible suppression of resistance. These data confirm that fluorinated analogues can simultaneously enhance antibiotic activity and constrain the evolutionary trajectories leading to resistance. This may be attributed to potentiation of fosfomycin by 3FG6P, which leads to lowering the mutant prevention concentration, thereby narrowing or closing the mutant selection window that would otherwise favor the amplification of pre-existing resistant mutants^53^. Another possibility is that the enhanced bactericidal kinetics by 3FG6P might minimize the temporal window for de novo mutations to arise. This rapid killing may also prevent the induction of mutagenic stress pathways, such as the SOS response, which are often triggered by antibiotic-induced damage and contribute to accelerated evolution^54^. Indeed, the enhanced bactericidal kinetics was observed in time-kill assays, which further underscored the clinical relevance of these fluorinated analogues, accelerating bacterial killing and sustaining suppression over 24 h. Sequencing of resistant isolates revealed that all carried IS1 insertions in *glpT* while *uhpT*, *uhpABC*, and *murA* remained intact (Fig. 5C, Supplementary Fig. S4). The preservation of wild-type *uhpT* suggests that sustained activation by 3FG6P creates strong selective pressure to maintain this pathway, restricting mutations that would compromise UhpT-mediated uptake. Because *glpT* disruption eliminates only one uptake route, such mutants remain rare, consistent with the dramatic suppression of resistance evolution we observe.

Critically, in the *Galleria mellonella* infection model, it could be seen that our in vitro data were translated into in vivo efficacy. While 3FG6P and 4FG6P did not show significant toxicity to the larvae, they showed improved survival and health outcomes as a potentiator of fosfomycin in the *E. coli* model (3FG6P) and the *S. aureus* model (4FG6P) over fosfomycin only or fosfomycin plus G6P. However, the combination of fosfomycin with 4FG6P provided less-superior protection to *G. mellonella* against *S. aureus* infections presumably due to comparative stability with G6P at 6 h of incubation (Fig. 3B, C). It is noteworthy that *E. coli* strain and *S. aureus* strain used in these experiments were multidrug-resistant strains, thus indicating the translational potential of FG6Ps.

Together, our findings highlight FG6Ps as promising adjuvants to restore and enhance fosfomycin susceptibility in clinically important pathogens, both Gram-negative and positive pathogens. By exploiting species-specific differences in transporter regulation and substrate recognition, these analogues provide a novel strategy to overcome an important resistance mechanism mostly associated with multidrug resistance, i.e., *fos*-mediated resistance, which has limited fosfomycin’s clinical utility. The species-specific efficacy patterns observed here (3FG6P optimal for *E. coli*, 4FG6P for *S. aureus*) also suggest that combination therapy with both analogues could provide broad-spectrum coverage for empiric treatment, while monotherapy with the species-optimized analogue would be more appropriate for directed therapy. Further development and optimization of these compounds could expand the arsenal against multidrug-resistant bacterial infections, with potential applicability across diverse pathogens.

The influx-targeting strategy demonstrated here focuses on the UhpT transporter system. Fosfomycin can also enter bacterial cells via the glycerol-3-phosphate transporter GlpT, whose expression is controlled by the GlpR repressor and induced by glycerol-3-phosphate, which inhibits GlpR DNA-binding activity^16^. Future studies could explore whether simultaneous activation of both UhpT and GlpT using combinations of metabolically stable signaling molecules could further enhance fosfomycin influx. However, basal GlpT levels have been reported to be sufficient for fosfomycin uptake^55^, suggesting that UhpT may be the rate-limiting transporter under most conditions, consistent with the dramatic potentiation we observe through UhpT activation alone.

This work establishes a compelling proof-of-concept advocating more studies in the uptake mechanism of antibiotic molecules, clearly demonstrating that understanding of the mechanism can offer opportunities to enhance uptake of antibiotic molecules into bacteria and overturn antimicrobial resistance of a certain mechanism. It is thus considered that more studies on the influx mechanism of antibiotics are warranted, and we believe that we have made a significant contribution to fight against microbial resistance.

In conclusion, this study establishes a compelling proof-of-concept for an “influx-targeting” strategy to combat antimicrobial resistance, demonstrating that the efficacy of legacy antibiotics can be revived by exploiting bacterial nutrient transport systems. We identified that fluorinated glucose-6-phosphate analogues (FG6Ps), specifically 3FG6P and 4FG6P, function as potent, metabolically stable adjuvants that robustly activate the UhpT-mediated transport pathway.

Unlike native G6P, which is rapidly catabolized, FG6Ps exhibit superior stability and enhanced binding affinity to sensor proteins such as HptA, leading to sustained upregulation of the influx machinery. Consequently, these analogues significantly potentiated fosfomycin activity, reducing MICs by up to 128-fold and restoring susceptibility in multidrug-resistant clinical isolates harboring *fosA* or *fosB* gene-mediated resistance. Furthermore, 3FG6P profoundly suppressed the emergence of resistant mutants under selective pressure and improved survival rates in a *Galleria mellonella* in vivo infection model.

Collectively, these findings suggest that targeting bacterial influx mechanisms offers a promising new paradigm for antimicrobial adjuvant development. By overwhelming resistance mechanisms through hyper-activation of antibiotic uptake, this approach could extend the clinical lifespan of fosfomycin and potentially other transport-dependent antibiotics against multidrug-resistant ESKAPE pathogens.

## Methods

### General materials

Unless otherwise stated, chemicals and biochemical reagents including enzymes were purchased from Merck Life Science. Media components were purchased from Merck Life Science or Melford Laboratories. All strains and primers used in the study are shown in Supplementary Tables S1 and S2, respectively.

### Synthesis and purification of fluorinated sugar phosphates

3-dexoy-3-fluoro- and 4-dexoy-4-fluoro-D-glucose-6-phosphates (3FG6P and 4FG6P, respectively, Biosynth Ltd.) were synthesized enzymatically following a previously reported method^40,41^. Briefly, 0.14 mmol of 3-dexoy-3-fluoro-D-glucose or 4-dexoy-4-fluoro-D-glucose was dissolved in triethanolamine buffer (TEA, 100 μM, pH 7.6) supplemented with KCl (5 μM) and MgCl₂ (10 μM). Phosphoenolpyruvic acid (1.1 equiv.) and ATP (0.01 equiv.) were added, and the pH was readjusted to 7.6. Reactions were initiated by addition of pyruvate kinase (660 U) and hexokinase (350 U). The mixture was incubated at 30 °C overnight. Typical reaction volumes were 1 mL.

Reaction progress was monitored by thin-layer chromatography (TLC). Upon completion, proteins were removed using centrifugal filtration (Amicon, MWCO 10,000; 1000 rpm), and the fluorinated sugar phosphates were purified by preparative TLC or reverse-phase HPLC as appropriate. The high-performance liquid chromatography (HPLC) system used was a Waters Infinity 1260, controlled by Waters Open Lab CDS software. Anion exchange chromatography was achieved on a semi-prep scale Waters Spherisorb® SAX column (250 mm × 10 mm, 5 μm). Solvents used in the mobile phase were as follows: solvent A – H_2_O; solvent B – 300 mM ammonium formate pH 3.5. A linear gradient (5 → 100% B, 40 min) was used, with a flow rate of 2.5 mL/min, a typical injection volume of 200 μL. Collected samples were subjected to LC-MS measurements, and fractions containing the desired product were pooled and lyophilized. The structure of 3FG6P and 4FG6P was verified through NMR and mass spectrometry. ^1^H, ^13^C, ^19^F and ^31^P NMR spectra were recorded on a BRUKER AV400 (400, 100, 376 and 162 MHz, respectively) spectrometers. HR mass spectra (MS) were recorded on a MaXis (Bruker Daltonics, Bremen, Germany) time of flight (TOF) mass spectrometer. Spectra are consistent with the published values^41^.

3-Fluoro-glucose-6-phosphate

**Figure:**
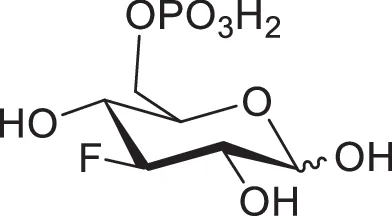

White powder, % yield (88%), α/β mixture (0.7/1). ^1^H NMR (400 MHz, D_2_O) δ 5.25 (app t. J = 3.8 Hz, 1 H, H1β), δ 4.69 (d, J = 6.8 Hz, 1 H, H1α) δ 4.60 (app. dt J = 53.1 Hz, J2,3 = J3,4 = 9.1 Hz, 1 H, H3α) δ 4.36 (app dt, J = 53.1 Hz, J = J = 9.1 Hz, 1 H, H3β), 4.09–3.74 (m, H2α, H2β, H5α, H5β, H6α, H6β, H6’α, H6’β), 3.64–3.51 (m, 2 H, H4α, H4β) ppm. ^13^C NMR (100 MHz, D_2_O) δ 96.18 (d, JC-F = 148.9 Hz, C3β), 95.32 (d, JC-F = 12.5 Hz, H1β), 94.39 (d, JC-F = 146.7, H3α), 92.3 (d, JC-F = 11 Hz, H1α), 73.93 (t, JC-F = 7.3 Hz, H5β), 72.67 (d, JC-F = 16.9 Hz, H2β) 72.05 (s, C5α) 70.19 (app. s H4β) 70.00 (d, JC-F = 16.9 Hz, H4α) 67.54 (d, JC-F = 17.6 Hz, C2α) 62.83 (s, C6β), 62.48 (s, C6α) ppm. ^19^F (376 MHz, D_2_O) δ −194.84, −199.71. ^31^P NMR (162 MHz, D_2_O) δ 4.04, 3.89 ppm. HRMS (ESI^-^), for C6H11FO8P [M-H]^-^, calcd. 261.0181, found 261.0179.

4-Fluoro-glucose-6-phosphate

**Figure:**
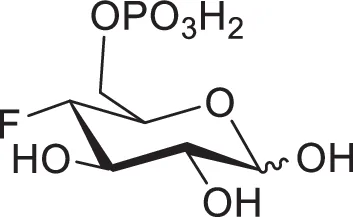

White powder. % yield (88%), α/β mixture. (0.8/1) ^1^H NMR (400 MHz, D_2_O) δ 5.21 (app. t, J1,2 = 3.5 Hz, 1 H, H1α), 4.68 (d, J1.2 = 8.1 Hz, 1 H, H1β), 4.39 (ddd, J4,F4 = 50.6 Hz, J3,4 = J4,5 = 6.5, H4α) 4.37 (ddd, J4,F4 = 50.5 Hz, J3-4 = J4,5 = 6.2 Hz, H4β), 4.16–3.64 (m, H, H3α, H3β, H5α, H5β, H6α, H6β, H6’α, H6’β) 3.53 (dd, 1 H, J1,2 = 6.5 Hz, J2,3 = 11.3 Hz, H2α) 3.31 (app. t, J1,2 = J2,3 = 9.4 Hz, 1 H, H2β) ppm. ^13^C NMR (100 MHz, D_2_O) δ 95.91 (d, JC-F = 1.5 Hz, H1β), 91.95 (d, JC-F = 1.5 Hz, H1α), 89.04 (d, JC-F = 179.0 Hz, H4β), 88.91 (d, JC-F = 181.2 Hz, H4α), 73.8 (d, JC-F = 17.6 Hz, H5β), 73.55 (d, JC-F = 8.8, H2β), 72.47 (d, JC-F = 16.1 Hz, H3β), 72.18 (d, JC-F = 25.7 Hz, H5α), 71.06 (d, JC-F = 17.06, H3α), 70.87 (d, JC-F = 8.1 Hz, H2α), 62.82 (app. dd, JC-F = 8.8 Hz, JC-P = 5.5 Hz C6β), 62.48 (s, C6α) ppm. ^19^F NMR (376 MHz, D_2_O) δ −197.62, −199.59 ppm. ^31^P NMR (162.0 MHz, D_2_O) δ 3.05 ppm. HRMS (ESI^-^) for C6H11FO8P [M-H]^-^, calcd. 261.0180, found 261.0181.

### Growth and culture conditions of bacterial strains

*Escherichia coli* strains were grown on Lysogeny broth (LB) agar (1.5% w/v) plates at 37 °C overnight unless otherwise noted. *Staphylococcus aureus* strains were grown on tryptic soy agar (TSA) plates at 37 °C overnight unless otherwise noted. Bacterial broth cultures were grown by inoculating LB broth media (BD Difco, USA) or tryptic soy broth (TSB) media (BD Difco, USA) with a single colony from plate. The bacterial broth cultures were grown under orbital shaking (200 rpm) culture conditions at 37 °C for 16–18 h. Bacterial cell growth was monitored by measuring optical density at 600 nm (OD_600_ of 1.0, equivalent to 1.0 × 10^9^ *S. aureus* USA300 colony-forming units (CFU/mL), 5.0 × 10^8^ *E. coli* NCTC12923 CFU/mL) using a spectrophotometer (GE Healthcare, United States). All the bacterial strains and plasmids used in the study are shown in Supplementary Table S1.

### Reporter system

The *uhpT* promoter–probe vectors were constructed with *mCherry* gene in the backbone of pACKK-P*gapA*-MCS vector (pACKK-P_*probe*_)^56^. The *uhpT* promoter of *E. coli* (P_*uhpT*_) was PCR-amplified using IP-*Pfu* DNA polymerase (Cosmogenetech, Korea). A linearized vector and a fragment were fused using Gibson Assembly Master Mix (New England Biolabs, USA). The pACKK-P_*probe*_) vector is a modified version of pRP1195 vector^57^, with multiple cloning sites (MCS) harboring P_*gapA*_ promoter which can serve as a positive control for reporter gene expression. The P_*gapA*_ promoter can be replaced using multiple flanking restriction endonucleases with the promoter of interest. The *uhpT* promoter of *E. coli* (P_*uhpT*_) sequence was obtained from the upstream region of *uhpT orf* of *E. coli* NCTC12923^42,58^ from the NCBI database. The P_*uhpT*_ promoter was PCR amplified from *E. coli* NCTC12923 genomic DNA using the primer pair P_*uhpT*_*_*fwd and P_*uhpT*_*_*rev (Supplementary Table S2), and assembled by replacing P_*gapA*_ promoter between *Pme*I*/SnaB*I in pACKK-P_*probe*_^56^, which contains *mCherry* gene along with *lambda* t0-terminator, to obtain pACKK-P_*uhpT*_*::mCherry* construct. For a control construct, pACKK-P_*uhpT-SUB*_*::mCherry*, a site-directed mutagenesis was performed in the P_*uhpT*_ promoter using the primer pair P_*uhpT-SUB*_*_*fwd and P_*uhpT-SUB*_*_*rev (Supplementary Table S2). These resultant constructs were used for the reporter assay to assess the induction of mCherry in response to glucose, G6P and its fluorinated analogue (3FG6P or 4FG6P).

### Reporter assay using fluorescence induction assay

The recombinant plasmid and the control vector for *E. coli* were electroporated into *E. coli* NCTC12923 using Gene Pulser X-cell (Bio-Rad, USA) at 750 V, 25 μF, 200 Ω resistance in 1 mm electroporation cuvette. The electroporated cells were recovered in 900 μL Terrific broth media (BD Difco, USA) for 1 h and plated on LB agar plates supplemented with 100 μg/mL carbenicillin (Glentham, USA). A single colony was picked and grown in LB media with 100 μg/mL carbenicillin. To assess the P_*uhpT*_ promoter activity, the *E. coli* NCTC12923 reporter strains were grown under orbital shaking (200 rpm) culture conditions at 37 °C for 16–18 h in M9 minimal medium with modifications to analyze the utilization of G6P analogs as the carbon source. Glucose was replaced with 0.4% (v/v) glycerol (hereafter referred to as M9-Gly) in the absence or presence of glucose, G6P, or a fluorinated analogue (3FG6P and 4FG6P). Overnight grown cultures were inoculated into media and grown until early-log phase (OD_600_ of 0.1). The culture was aliquoted at 200 μL in a 96-well black plate with clear flat bottoms (Corning, USA), supplemented with glucose, G6P, or FG6P at the final concentration of 50 μM. The fluorescence intensity and bacterial growth (OD_600_) were recorded after 7.5 h and 9 h using Spark 10 M Multimode Microplate Reader (Tecan, Switzerland). The fluorescence signal was normalized with OD_600_ at their corresponding fluorescence signals. The bacterial strain harboring the control vector was used as a control to assess the background signal. All assays were performed in triplicates in at least two independent biological replicates.

### G6PDH assay

*E. coli* BL21(DE3) cells were cultured overnight in LB broth at 37 °C with shaking (200 rpm). For assays, LB medium (10 mL) supplemented with G6P or a fluorinated analogue (50–100 μM) was inoculated with a 1:2000 dilution of the overnight culture. At designated time points, cells were pelleted by centrifugation (13,000 × *g*, 2 min), and 500 μL of the supernatant was combined with reaction buffer (50 mM Tris-HCl, pH 7.6, 10 mM MgCl₂, 1.4 mM NADP⁺, and 1–2 U/mL glucose-6-phosphate dehydrogenase from *Leuconostoc mesenteroides*). Substrate concentrations were quantified spectrophotometrically by monitoring NADPH formation at 340 nm at 25 °C. Calibration curves were generated using G6P/FG6P standards (5–100 μM).

For kinetics, the same reaction condition was used with varying concentrations of G6P, 3FG6P, or 4FG6P (5–200 µM) in the presence of NADP^+^ (1.4 mM), and NADPH production was monitored spectrophotometrically at 340 nm over 10 min at 25 °C. Initial rates were determined from the linear portion of the reaction progress curves and plotted against substrate concentration. Data were fitted to the Michaelis-Menten equation by nonlinear regression using SciPy (v1.17.1) in Python. K_m_ and V_max_ values were reported as best-fit estimates ± standard error.

### Cell-suspension assay for fosfomycin uptake using LC-MS analysis

A single colony of *E. coli* ATCC8739 was inoculated in 5 mL M9 medium and grown overnight at 37 °C. The overnight grown culture was washed with 0.9% saline once and resuspended into 5 mL M9 medium supplemented with 0.4% glycerol. A 400 mL of M9 medium supplemented with 0.4% glycerol was inoculated at 1:100 dilution and allowed to grow until OD_600_ of 1.0–1.2. An 80 mL culture was prepared in 250 mL flasks containing M9 medium with 0.4% glycerol and divided into four conditions: growth control, fosfomycin (8 µg/mL), fosfomycin (8 µg/mL) plus G6P (50 µM), and fosfomycin (8 µg/mL) plus 3FG6P (50 µM). Bacterial growth was monitored at 0, 1, 2, and 4 h, and aliquots were collected at 2 and 4 h time points for LC-MS analysis. The collected cells were centrifuged at 4000 rpm (4 °C), and the cell pellets were washed twice using 10 mL of 0.9% saline. It is noteworthy that the residual saline was completely removed to avoid contamination of any extracellular fosfomycin. One mL of chilled methanol was added to the cell pellets, which were then vortexed vigorously, followed by two 30-min freeze-thaw cycles (from −80 °C to room temperature). Finally, the samples were centrifuged at maximum speed (17,000 × *g*), and the methanolic extract of intracellular fosfomycin was collected into new vials for further LC-MS analysis.

The LC-MS analysis was performed on an Agilent 1260 Infinity III HPLC system coupled with an Agilent InfinityLab Pro iQ single quadrupole mass spectrometer equipped with an ESI ion source. Fosfomycin was separated using acetonitrile containing 0.1% formic acid (organic phase) and 0.1% formic acid in water (aqueous phase) as solvents on a Hector C18 column (250 mm × 4.6 mm, particle size 5 µm) at a column temperature of 25 °C. The gradient started at 1% organic for 3 min after injection and increased linearly to 100% organic over 27 min, then 100% organic for 3 min, and down to 1% organic for 10 min at a flow rate of 0.700 mL/min. A water stock solution of fosfomycin standard was prepared and diluted at four different concentrations (0.5, 1, 2, and 4 µg/mL) for the construction of calibration curves. The resulting mixtures and sample extracts were analyzed using the LC-MS conditions described above. Quantification was performed by peak area integration using Agilent OpenLAB CDS. The identity of the extracted intracellular fosfomycin was analyzed by mass spectrometry wherein the capillary voltage 3500 V, gas temperature 325 °C, gas flow 13 L/min, nebulizer pressure 55 psi, fragmentor voltage 100–130 V. Each sample was analyzed in negative ionization mode using a *m/z* range of 100 to 400.

### Antimicrobial susceptibility testing

Minimum inhibitory concentrations (MICs) were determined in triplicate following the broth microdilution method described in the Clinical and Laboratory Standards Institute (CLSI) M7. Overnight cultures were adjusted to a 0.5 McFarland standard (*c*. 1–2 × 10⁸ CFU/mL) and diluted to yield an initial inoculum of *c*. 5 × 10⁵ CFU/mL. Assays were performed in cation-adjusted Mueller–Hinton broth (CAMHB) with fosfomycin concentrations ranging from 0.03 to 64 mg/L, supplemented with G6P or a fluorinated analogue (50 μM). In case of MICs for FG6P only, the concentrations ranged from 0.1 to 256 mg/mL. Inoculum densities were verified by plating 10⁻⁴ and 10⁻⁵ dilutions of growth controls on non-selective agar. MIC values were defined as the lowest concentration of fosfomycin that inhibited visible growth.

### Time-kill assay

Time-kill kinetics of fosfomycin were assessed in cation-adjusted Mueller–Hinton broth (CAMHB) broth in the absence or the presence of 50 µM G6P or an FG6P. Cultures of *E. coli* (DH5α) or BL21(DE3) were adjusted to ~1 × 10⁶ CFU/mL and exposed to fosfomycin at 0.5–4× MIC. Samples were withdrawn at 0, 0.5, 1, 2, 4, 6, and 24 h, serially diluted in sterile broth, and plated on Mueller–Hinton II agar for CFU enumeration following 24 h incubation. Growth controls (no antibiotic) and sterility controls (medium only) were included in each experiment. All conditions were performed in triplicate.

### Mutation frequency assay

The frequency of fosfomycin-resistant mutants was determined following serial passage under selective pressure. *E. coli* DH5α was cultured in LB broth at 37 °C with shaking (200 rpm) and adjusted to ~10⁷ CFU/mL. Populations were serially passaged for seven consecutive days in LB medium containing fosfomycin at 0.25×, 2×, or 8× MIC, with or without supplementation of 50 µM G6P or 50 µM 3FG6P. Controls were passaged in drug-free medium. At each 24-h interval, cultures were diluted 1:1000 into fresh medium of the same composition.

At day 7, bacterial suspensions were plated in parallel on LB agar containing fosfomycin at 16× or 32× MIC, and on drug-free plates to determine total viable counts. Colonies arising after 24 h were enumerated, and mutation frequencies were calculated as the ratio of resistant CFU to total CFU. All conditions were tested in biological triplicate.

### Quantitative reverse‑transcription polymerase chain reaction (qRT‑PCR) analysis

Overnight cultures grown in minimal media with glycerol as a carbon-source were inoculated to OD_600_ of 0.1 and grown till mid-log phase. Bacteria were treated with G6P or an FG6P at 50 μM for 15 min and were harvested by centrifuging at 5000 × *g* at 4 °C. Total RNA was isolated using RNAeasy mini kit (Qiagen, Germany) according to the manufacturer’s protocol. One μg of RNA was treated with 1 unit of RNase-fee DNase I (Sigma-Aldrich, USA) at room temperature for 15 min, followed by addition of stop buffer and heat-inactivation of DNase I at 70 °C for 10 min. The cDNA was synthesized using a random hexamer premix, RNA to cDNA EcoDry premix (Takara Bio, Japan). Quantitative qRT–PCR was performed using Taq Pro Universal SYBR qPCR Master Mix (Vazyme, China) at annealing temperature of 55 °C. The *gyrA* gene was used as a housekeeping gene and the primers used pr qRT–PCR are listed in Supplementary Table S2. Relative gene expressions were calculated by normalization using 2^−ΔΔCT^ method. Three independent biological replicates were prepared in the same plate, and three technical replicates were carried out. The statistical significance was calculated by Student’s *t*-test (*p* < 0.05).

### *Galleria mellonella* infection model

*E. coli* NCTC12923 and *S. aureus* USA300 isolates were grown overnight in media under orbital shaking (200 rpm) culture conditions at 37 °C. 100-fold dilution of the overnight cultures were inoculated into media and grown till mid-log phase (OD_600_ of 1.0). Bacterial cells were collected by centrifugation (3220 × *g*, 4 °C) and washed once with 0.9% Saline. Cell number was maintained by adjusting the optical density (OD_600_) to 1 in 0.9% Saline. A *Galleria mellonella* (wax moth) infection model was used as described earlier^59^, with modifications. Briefly, *G. mellonella* larvae (Sworm, Korea) were stored for one day and utilized within two days of their receipt. Ten wax moth larvae of similar size (2 ± 0.2 cm, *n* = 10) were chosen for each group. Wax-worms were injected with 20 μl of bacteria in the left hindmost proleg, followed by fosfomycin (Glentham, USA) and G6P or an FG6P in the right hindmost proleg using a 0.3 mL syringe (Becton Dickinson and Company, USA) and kept at 37 °C for observation^60–63^. Survival rate and health-index were calculated based on activity, cocoon formation, melanization, and survival at different time intervals^62^ and the experiment was terminated within 72 h of injection.

### Isothermal titration calorimetry (ITC) assay

#### Expression and purification of HptA

The HptA amino acid sequence (UniProt X5DVD1) without the first 20 amino acids (signal sequence) was used to design the plasmid, which was codon-optimized for expression in *E. coli* and synthesized by Genscript (Piscataway, NJ, USA). The gene was supplied in the vector pET21b, which was coded as pET21b-HptA. The plasmid was transformed into *E. coli* BL21 (DE3) strain for expression. The recombinant bacteria cells were cultured in LB medium at 37 °C to reach an OD_600_ of 0.6 and then induced with 1 mM isopropyl-b-D-thiogalactoside (IPTG) for 4 h at 37 °C. The cells were harvested and lysed by sonication in buffer A (20 mM Tris-HCl pH 8, 500 mM NaCl, 40 mM imidazole). The lysate was centrifuged at 20,000 × *g* for 1 h and the supernatant was loaded onto a Ni-NTA column (GE Healthcare). After washing with buffer A, the protein was eluted from the column using imidazole gradient in buffer B (20 mM Tris-HCl pH 8, 500 mM NaCl, 1 M imidazole). The protein was desalted with PD-10 desalting column (GE Healthcare, Chalfont St Giles, UK) using buffer C (20 mM Tris-HCl pH 8, 150 mM NaCl) and loaded onto a AIEX column (GE Healthcare, Chalfont St Giles, UK) for further purification. Purity improved HptA was eluted when the column was washed with buffer C. The fractions were collected for ITC assay.

#### Isothermal titration calorimetry (ITC) assay

ITC assays were conducted using a MicroCal iTC200 calorimeter (GE Healthcare, Chalfont St Giles, UK) in buffer C at 25 °C. HptA at concentration of 39–40 μM (39 µM for the binding assay with glucose and 40 μM for the binding assay with other ligands) was added into the cell. The syringe was filled with ligands at a concentration of 700–800 μM (800 μM of glucose and 700 μM of other ligands). 2 μL aliquots of a ligand (G6P, 4FG6P or glucose) were sequentially injected 19 times into the cell with a spacing interval of 120 s after each injection. The reference used as a baseline was obtained by measurement of cell filled with the buffer and a syringe filled with the ligand. Data was fitted and analyzed using a one-site binding model.

## Supplementary information

Supplementary materials

## Data Availability

All data generated or analyzed during this study are included in this published article and the supplementary information file. The raw data and materials in the study are available from the corresponding authors on reasonable request.
