# Supplementary material for: Targeting bacterial influx pathways to defeat antibiotic resistance: fluorinated glucose-6-phosphate analogues revive fosfomycin efficacy
Source: NPJ Antimicrob Resist. 2026 Jun 21;4:72. doi: 10.1038/s44259-026-00239-y (PMC13745569; doi:10.1038/s44259-026-00239-y)
Supplement: Supplementary file 1 [file 44259_2026_239_MOESM1_ESM.docx]

Supplementary Table S1. Bacteria strains and plasmids used in this study

| **Strain/Plasmid** | **Description** | **Source** |
| --- | --- | --- |
| Bacteria |  |  |
| *Escherichia coli* |  |  |
| DH5α | cloning strain | Lab collection |
| BL21(DE3) | protein expression host strain | Novagen, USA |
| NCTC12923 | ATCC 8739, KCTC 2571 | KCTC, Korea |
| A004 | UTI clinical isolate harboring fosA^1^ | Lab collection |
| *Staphylococcus aureus* |  |  |
| USA300 | wild-type epidemic community-associated methicillin-resistant isolate FPR3757 LAC strain | NARSA^2^ |
| COL | MRSA isolate containing fosB ^3^ | Lab collection |
| Plasmid |  |  |
| pACKK-P*_gapA_*-MCS | synthetic MCS with P*_gapA_* promoter cloned at *Eag*I*-Bam*H1 sites | ^4^ |
| pACKK-P*_gapA_*::*mCherry* | mCherry protein expressing under P*_gapA_* promoter; *Amp^r^* and *Cm^r^* | ^4^ |
| pACKK-P*uhpT::mCherry* | mCherry expressing under P*uhpT* promoter; *Amp^r^* and *Cm^r^* | This study |
| pACKK-P*_uhpT-SUB_::mCherry* | mCherry expressing under site-directed mutation of P*_uhpT-SUB_* promoter; *Amp^r^* and *Cm^r^* | This study |
| pET21b-HptA | carrying a native gene that encodes HptA in fusion with a C-terminal 6×His tag; *Amp^r^* | This study |

Supplementary Table S2. Primers used for the construction of reporter system and primers used in qRT-PCR analyses.

| **Name** | **Sequence** (5’ **→** 3’) |
| --- | --- |
| P*_uhpT__*fwd | ctagccgccggcggtttaagcgtgatgcatctcacc |
| P*_uhpT_*_rev | ccttgctcaccatctcgagtacggcatgcattgtaaaaatc |
| P*_uhpT-SUB_*_fwd | gctatctcccatgggatttgctgctgatttttac |
| P*_uhpT-SUB_*_rev | gtccaggaaaaacctgga |
| EC_*gyrA*_fwd | gaactgttgcgtattcttgg |
| EC_*gyrA*_rev | gatacttaacgtagccctgg |
| EC_*uhpA*_fwd | gccggatattgccattaaac |
| EC_*uhpA*_rev | agctctacgtcgttactgac |
| EC_*uhpB*_fwd | attaccgttattgcctgctt |
| EC_*uhpB*_rev | ttagccagtaaatcagcagc |
| EC_*uhpC*_fwd | gtatatctggctgctttcgt |
| EC_*uhpC*_rev | tgccgttaaacaatttgtcc |
| EC_*uhpT*_fwd | caaattacctgttcgatggc |
| EC_*uhpT*_rev | gccacttggtcatatcggta |
| *uhpT*-flanking-PCR_fwd | taaccagtcaggggctatttgatggcg |
| *uhpA*-flanking-PCR_rev | gcattctttgtctgccgattcaggac |
| *uhpT*-Seq-1_fwd | ttcaaacagcgtaaagccctg |
| *uhpT* Seq-2_fwd | gtgatggagaaacccagg |
| *uhpC*-Seq-3_fwd | atggtgaagaagcaggttg |
| *uhpC*-Seq-4_fwd | cggtacgtgaataccagg |
| *uhpB*_Seq-5_fwd | atcgattcgccattcgagatg |
| *uhpB*_Seq-6_fwd | acaccagacatatcgggg |
| *uhpA*-Seq-7_fwd | tatccggcgtcagataacag |
| *murA*-flanking-PCR_fwd | atcgacagaacgcagttgatgcg |
| *murA*-flanking-PCR_rev | cgatcaaagcgtatacccctgc |
| *murA*_Seq-1_rev | cgcagccagatactctcgac |
| *murA*_Seq-2_fwd | ttcgatgacgatacgatcgg |
| *glpT*-flanking-PCR_fwd | tatccactggcaccgcgatgggcg |
| *glpT*-flanking-PCR_rev | aataatgtgtgcggcaattcac |
| *glpT*-Seq-1_fwd | aaggaagtaggcccaggagg |
| *glpT*-Seq-2_fwd | acaaacatcaccgcaatgctc |

**Supplementary Table S3. Mutational frequencies of E. coli DH5α following 7-day serial passage with fosfomycin ± adjuvants.**

| **Condition** | **16 x MIC (mean ± SD)** | **32 x MIC (mean ± SD)** |
| --- | --- | --- |
| Growth control (GC) | 6.69 × 10⁻¹² ± 5.85 × 10⁻¹² | 1.32 × 10⁻¹⁵ ± 9.46 × 10⁻¹⁶ |
| 8 x MIC fos | 2.57 × 10⁻¹ ± 2.53 × 10⁻¹ | 7.99 × 10⁻² ± 7.82 × 10⁻² |
| 2 x MIC fos | 3.78 × 10⁻² ± 3.31 × 10⁻² | 2.32 × 10⁻² ± 3.40 × 10⁻² |
| 0.25 MIC fos | 1.29 × 10⁻⁶ ± 7.07 × 10⁻⁷ | 2.50 × 10⁻⁸ ± 2.97 × 10⁻⁸ |
| 8 x MIC fos + 50 µM G6P | 1.71 × 10⁻¹ ± 2.27 × 10⁻¹ | 2.46 × 10⁻² ± 2.70 × 10⁻² |
| 2 x MIC fos + 50 µM G6P | 3.29 × 10⁻³ ± 4.35 × 10⁻³ | 2.16 × 10⁻³ ± 3.26 × 10⁻³ |
| 0.25 MIC fos + 50 µM G6P | 6.44 × 10⁻⁶ ± 5.49 × 10⁻⁶ | 1.66 × 10⁻⁵ ± 2.41 × 10⁻⁵ |
| 8 x MIC fos + 50 µM 3FG6P | 3.93 × 10⁻⁴ ± 3.07 × 10⁻⁴ | 6.61 × 10⁻⁴ ± 5.68 × 10⁻⁴ |
| 2 x MIC fos + 50 µM 3FG6P | 1.94 × 10⁻⁵ ± 1.77 × 10⁻⁵ | 7.41 × 10⁻⁵ ± 8.42 × 10⁻⁵ |
| 0.25 MIC fos + 50 µM 3FG6P | 5.01 × 10⁻⁸ ± 4.67 × 10⁻⁸ | 1.51 × 10⁻⁹ ± 1.20 × 10⁻⁹ |

**Supplementary Figure S1.** Schematic diagram showing the synthesis of fluorinated glucose 6-phosphate from fluorinated glucose using ATP-dependent coupled reaction of hexokinase (HK) and pyruvate kinase (PK). HK utilized the ATP to convert fluorinated glucose to fluorinated glucose 6-phosphate and ADP. The high-energy phosphate group from phosphoenolpyruvate (PEP) is transferred to ADP to generate one molecule of ATP and pyruvate.

B

A

D

C

**Supplementary Figure S2.** Enzyme kinetics of glucose-6-phosphate dehydrogenase

with native and fluorinated substrates. Data points represent mean ± SD of three technical replicates. Lines represent best-fit to Michaelis-Menten equation. (A) glucose-6-phosphate (G6P), (B) 3-fluoro-glucose-6-phosphate (3FG6P), (C) 4-fluoro-glucose-6-phosphate (4FG6P) (D) Summary of kinetic parameters. Values represent mean ± SD from three independent experiments.

**Supplementary Figure S3.** Cell-suspension assay for fosfomycin antibiotic uptake wherein various steps showing the cell-suspension assay of fosfomycin. (**A**) Schematic diagram showing the steps of cell suspension assay to estimate the intracellular concentration of fosfomycin in *E. coli* ATCC8739 (*E. coli* NCTC12923). (1) The overnight grown culture was washed with 0.9% saline once and resuspended into 5 mL M9 medium supplemented with 0.4% glycerol. (2) A 400 mL of M9 medium supplemented with 0.4% glycerol was inoculated at 1:100 dilution and allowed to grow until OD_600_ of 1.0–1.2. (3) An 80 mL culture was prepared in 250 mL flasks containing M9 medium with 0.4% glycerol and divided into four conditions: growth control, (A) fosfomycin only (8 µg/mL), (B) fosfomycin (8 µg/mL) plus G6P (50 µM), and (C) fosfomycin (8 µg/mL) plus 3FG6P (50 µM). (4) Bacterial growth was measured at each time-points (0‒4h) to normalize the fosfomycin from each sample. (5) Bacterial samples (10 mL) were taken 2, and 4 hours’ time points for LC–MS analysis to measure the intracellular content of fosfomycin. *Image created in BioRender.* (**B**) Calibration curve of fosfomycin showing the relationship between area under curve (AUC) and fosfomycin.

**Supplementary Figure S4.** Disruption of *glpT* gene due to insertion of IS1 element (768 bp). (**A**) Isolates exposed to fosfomycin showed an increase in the *glpT* gene as compared to the *glpT* from the *E. coli* DH5α transferred continuously without fosfomycin during adaptive laboratory evolution. (**B**) Site of insertion of IS1 in *glpT* gene, and (**C**) nucleotide sequence of inserted IS1 harboring *InsA* and *InsB* orfs with (A)_6_C tract.

**Supplementary Figure S5.** Quantitative reverse-transcription polymerase chain reaction (qRT–PCR) analysis to evaluate the expression of *uhpA*, *uhpB*, *uhpC*, and *uhpT* in *E. coli* NCTC12923 in the presence of compound (glucose, G6P and 3FG6P). (**A**) The treatment of 3FG6P led to marked upregulation of all four genes, with fold changes of 4.2 (*uhpA*), 6.0 (*uhpB*), 6.9 (*uhpC*), and 46.4 (*uhpT*) relative to untreated controls in M9 minimal medium supplemented with glycerol (M9-Gly). Notably, the greatest induction was observed for *uhpT*, consistent with its role as the primary fosfomycin transporter. (**B**)

In M9 medium supplemented with both glycerol and glucose, a similar transcriptional profile was observed, with 3FG6P inducing *uhpA*, *uhpB*, *uhpC*, and *uhpT* by 49.0-, 54.2-, 1.9-, and 22.2-fold, respectively; wherein the expression levels of *uhpA*, *uhpB*, and *uhpT* in response to 3FG6P were significantly elevated compared to G6P.

**Supplementary Figure S6.** Optimization Infection parameters by inoculating larvae with varying colony-forming units (CFUs). (a) *E. coli* NCTC12923; (b) *S. aureus* USA300, by monitoring survival over 72 hours.

**Supplementary Figure S7.** Comparative analysis of 3FG6P as a potentiator in *S. aureus* USA300. Based on survival (A) and health-index (B) analyses, it protects ~80% larvae but at higher concentration (16 µg/mL) of fosfomycin, which presumably shows species-specificity. Data is presented as the means ± s.d. of the means. Statistical comparisons among groups were conducted using one-way ANOVA followed by a post-hoc Tukey's test. Differences were considered statistically significant for *p* values < 0.05 and labeled in the graph as follows: *ns*, nonsignificant; *, *p* ≥ 0.05; and **, *p* ≤ 0.01, and ***, *P <* 0.001.

**B**

**A**

**a**

**b**

**Supplementary Figure S8.** Glucose was kept as negative control in isothermal titration calorimetry (ITC) experiments to show the preferred binding of 4-fluoro-glucose-6-phosphate (4FG6P) and glucose-6-phosphate (G6P) with staphylococcal HptA. (a) Representative ITC thermograms and binding isotherms for the interaction of HptA with glucose, and (b) While the dissociation constant (*K_D_*) for 4FG6P was determined to be 0.36 μM, compared to 2.70 μM for G6P, indicating a strong preference for phosphorylated hexoses, glucose showed negligible interaction and binding with staphylococcal HptA.

REFERENCES

1	Seok, H. *et al.* Fosfomycin resistance in *Escherichia coli* isolates from South Korea and *in vitro* activity of fosfomycin alone and in combination with other antibiotics. *Antibiotics* **9**, 112 (2020). https://doi.org/10.3390/antibiotics9030112

2	Fey, P. D. *et al.* A genetic resource for rapid and comprehensive phenotype screening of nonessential *Staphylococcus aureus* genes. *mBio* **4**, e00537-00512 (2013). <https://doi.org/10.1128/mBio.00537-12>

3	Gill Steven, R. *et al.* Insights on evolution of virulence and resistance from the complete genome analysis of an early Methicillin-Resistant *Staphylococcus aureus* strain and a biofilm-producing Methicillin-Resistant *Staphylococcus epidermidis* strain. *J Bacteriol* **187**, 2426-2438 (2005). <https://doi.org/10.1128/jb.187.7.2426-2438.2005>

4	Sultan, M. *et al.* Targeting the G-quadruplex as a novel strategy for developing antibiotics against hypervirulent drug-resistant *Staphylococcus aureus*. *J Biomed Sci* **32**, 15 (2025). <https://doi.org/10.1186/s12929-024-01109-3>
